# NEArender: an R package for functional interpretation of ‘omics’ data via network enrichment analysis

**DOI:** 10.1186/s12859-017-1534-y

**Published:** 2017-03-23

**Authors:** Ashwini Jeggari, Andrey Alexeyenko

**Affiliations:** 1 National Bioinformatics Infrastructure Sweden, Science for Life Laboratory, Stockholm, Sweden; 2 Department of Microbiology, Tumor and Cell biology, Karolinska Institutet, Stockholm, Sweden; 3 Department of Cell and Molecular Biology, Karolinska Institutet, Stockholm, Sweden

**Keywords:** Enrichment, Network analysis, Network benchmark, R package

## Abstract

**Background:**

The statistical evaluation of pathway enrichment, i.e. of gene profiles' confluence to the pathway level, allows exploring molecular landscapes using functionally annotated gene sets. However, pathway scores can also be used as predictive features in machine learning. That requires, firstly, increasing statistical power and biological relevance via a network enrichment analysis (NEA) and, secondly, a fast and convenient procedure for rendering the original data into a space of pathway scores. However, previous implementations of NEA involved multiple runs of network randomization and were therefore slow.

**Results:**

Here, we present a new R package NEArender which can transform raw 'omics' features of experimental or clinical samples into matrices describing the same samples with many fewer NEA-based pathway scores. This is done via a parametric estimation of the null binomial distribution and is thus much faster and less biased than randomization procedures. Further, we compare estimates from these two alternative procedures and demonstrate that the summarization of individual genes to pathways increases the statistical power compared to both the default differential expression analysis on individual genes and the state-of-the-art gene set enrichment analysis. The package also contains functions for preparing input, modeling null distributions, and evaluating alternative versions of the global network.

**Conclusions:**

Beyond the state-of-the-art exploration of molecular data through pathway enrichment, score matrices produced by NEArender can be used in larger bioinformatics pipelines as input for phenotype modeling, predicting disease outcomes etc. This approach is often more sensitive and robust than using the original data. The package NEArender is complementary to the online NEA tool EviNet (https://www.evinet.org) and, unlike of the latter, enables high performance of computations off-line.

The R package NEArender version 1.4 is available at CRAN repository

https://cran.r-project.org/web/packages/NEArender/

**Electronic supplementary material:**

The online version of this article (doi:10.1186/s12859-017-1534-y) contains supplementary material, which is available to authorized users.

## Background

NEA employs network topology to evaluate functional impact of experimentally determined genes and gene sets by detecting enrichment of previously characterized gene sets, such as pathways. NEA became a natural extension of the gene set enrichment analysis, GSEA [1] into the network domain [2, 3]. Performance and applicability of GSEA have been limited by the following: 1) only a minority of genes possesses specific pathway annotations, 2) it can be applied only to genes altered in a specific way, detectable by the given platform (typically transcriptomics) and not to those regulated via other mechanisms, and 3) statistical power of the analysis is limited by gene set size. NEA largely overcomes these limitations due to one key difference: while GSEA counts the number of genes shared between an experimental list and a pathway, NEA considers network edges between any genes of the two sets in the global network. On the other hand, the earlier NEA versions had an own drawback: the error rate was estimated in multiple, time-consuming instances of randomized networks. Being a non-parametric approach, the randomization allows solving a wide range of higher-order topological problems. However, for many applications this would be prohibitively slow. In the present R package, we implement a much faster parametric estimation of connectivity rate expected by chance and show that this also eliminates bias on small gene sets. Finally, we provide an illustration of how NEA increases robustness of non-replicated analyses of gene differential expression.

## Results

### Software functionality

NEArender possesses both core and ancillary functions for network enrichment analysis. It is implemented as an R package and described in details, beyond the official manual, in an R vignette https://cran.r-project.org/web/packages/NEArender/vignettes/NEArender_vignette.pdf

**Fig. 1:**
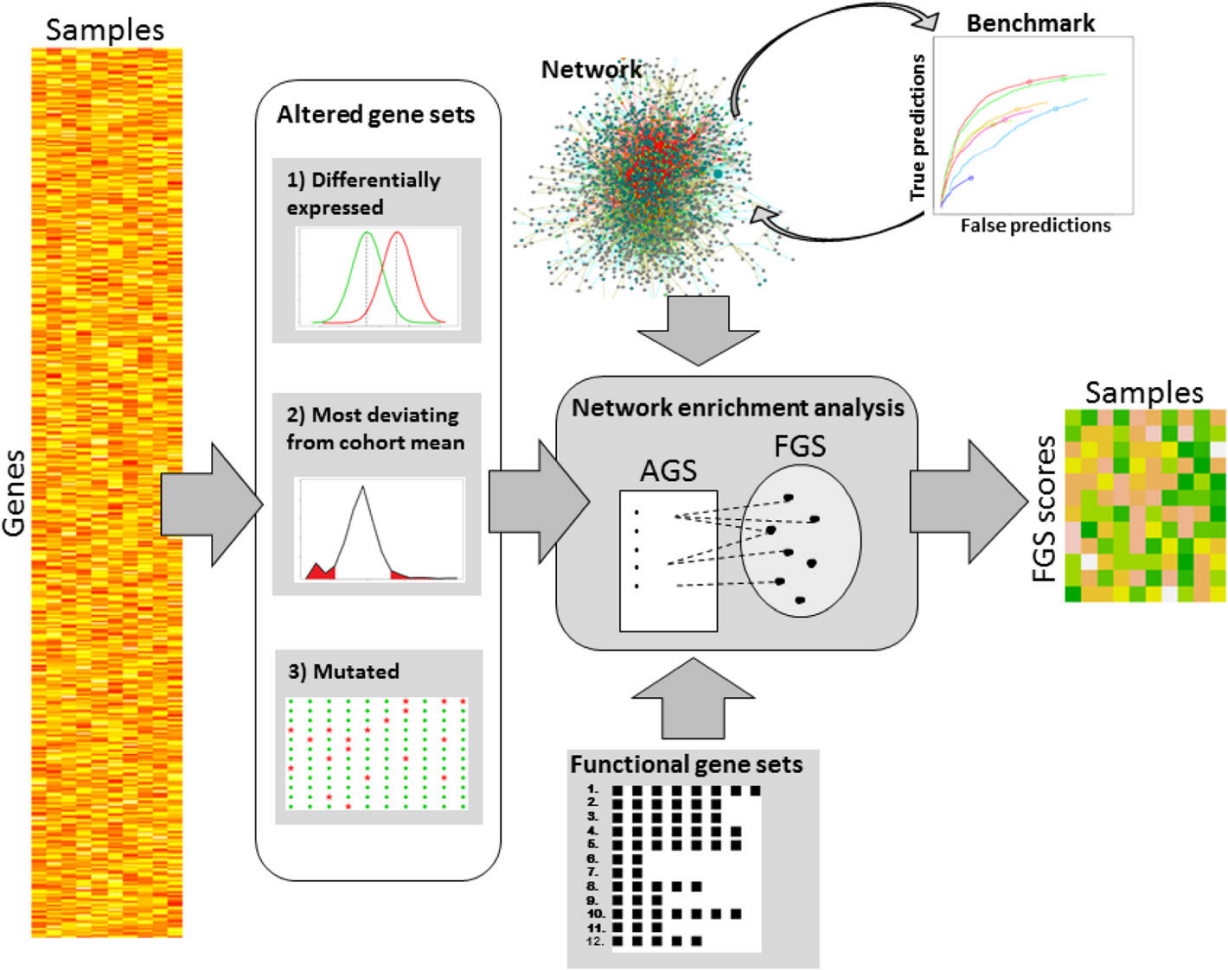
Analysis flow in NEArender. The original matrix of 'omics' (mutation, methylation, expression etc.) data described a limited number of samples (patients etc.) with a much larger number of gene feature rows. At the first, preparatory step each sample was described via a characteristic sample-specific altered gene set (AGS). In parallel, a collection of functional gene sets (FGS) that share certain functionally annotations (within each set) was downloaded or prepared otherwise. A global gene/protein network (NET) was also provided (possibly selected from a number of alternatives based on benchmark results). In the course of network enrichment analysis (NEA) each AGS received as many NEA scores as there were FGSs, i.e. obtained coordinates in the multidimensional FGS space. This created an output matrix of the same number of sample columns but many fewer rows

The input shall contain three components: 1) one or multiple user-defined (experimental or theoretical) gene sets which have to be functionally characterized, called altered gene sets (AGS); 2) a collection of functional gene sets (FGS) which would enable functional characterization through their known functions, and 3) a global network of functional coupling (NET). Input can be provided as either text files or pre-processed R lists and matrices. Figure 1 presents the relationship between the major components and steps of NEArender.

In fact, although we denote input in terms of “genes”, a range of functional nodes of a biological network can be analyzed with this algorithm, such as protein molecules, genomic regions that encode proteins, microRNAs, promoters, and enhancers etc. Nodes listed in AGS, FGS, and NET should employ the same ID format. The package performs network enrichment analysis through the fast binomial test as described in Methods and quantifies enrichment in each AGS-FGS pair with a number of statistics: chi-squared score, z-score, *p*-value, q-value (the *p*-value adjusted for multiple testing, a.k.a. false discovery rate [4], number of network edges that exist between any nodes of AGS and FGS (but neither within AGS nor within FGS), and respective number of AGS-FGS edges expected by chance, calculated with the binomial formula.

In order to run enrichment analysis, the user should prepare the input components. Since the AGS is the most dynamic and user-specific part of the input, functionality for AGS compilation and processing is most developed. First, AGSs can be prepared in advance externally, e.g. as a list of genomic variations reported in a given genome. Alternatively, the package functions can create AGSs from sample columns of an R matrix. This can be done with a number of different algorithms (two of these we use below in the section ‘Robustness of results obtained from different replicates’. Then the number of genes included in each AGS would be either data-driven (all significant genes) or pre-defined by user (top ranking ones regardless of significance). In addition, the algorithm ‘toprandom’ generates random AGSs of a user-set size. Finally, a special function allows direct creation of AGSs as full sample-specific sets of mutated genes. FGSs are usually imported from a file by listing all members of each functional set. Due to the high network density and, hence, statistical power of NEA, there is a special option, which is not available in GSEA: single genes can be treated as FGS as well. A full list of such single-gene FGSs can be created from all network nodes of NET with parameter as_genes_fgs, so that each FGS item in the output list contains just one gene. It is practical to use a large pre-compiled FGS collection, such as all ontology terms, pathways, or a union of resources, e.g. MSigDB database [5]. Alternatively, users can create custom single- or multi-gene FGS collections of their own. We note however that using relatively few FGSs and/or AGSs in one analysis (so that the total number of AGS-FGS tests is below a few hundreds) would not allow estimating the q-values properly.

While higher order topological biases are, as discussed above, of arguable importance for NEA, another network feature is vital for the enrichment evaluation used in this package. Namely, the parametric algorithm would produce unbiased estimates only in scale-free networks, i.e. where node connectivity values follow the power law distribution [6]. Being an almost ubiquitous feature in the full scale biological networks, this is still not the case in networks that are artificially constructed from e.g. ChIP-seq based collections of transcription factor binding events [7] or from computationally predicted microRNA-transcript targeting data [8]. If such network components are desirable, we recommend employing software that involves network permutation tests, i.e. the randomization, such as in [2, 3]. Therefore, the package enables evaluating network topology for scale-freeness and second-order dependencies (described below) as well as benchmarking alternative NETs using either standard or custom FGSs, as described in [9]. Briefly, the benchmark consists of as many test cases as there are FGS members in total (multiple occurrences of the same genes in different FGS are treated separately). For each such gene, the procedure tests the null hypothesis of the gene not being an FGS member. The true positive or false negative result is assigned if the gene receives an NEA score above or below a certain threshold, respectively. In parallel, randomly picked genes with close node degree values are tested against the same FGS to estimate specificity via the false positive versus true negative ratio. The counts of alternative test outcomes TP, TN, FP, and FN at variable NEA thresholds are used for plotting ROC curves.

### Comparison to randomization based algorithm

**Fig. 2:**
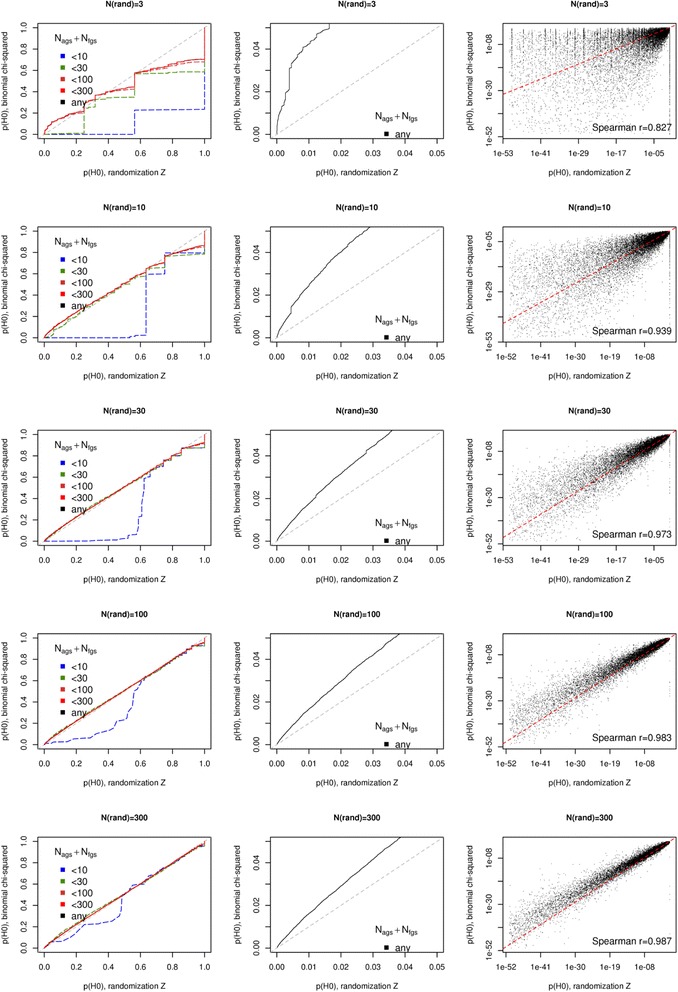
Comparative sensitivity and sources of bias in randomization-based versus binomial calculation of network enrichment. Network enrichment between all vs. all 330 gene sets was analyzed with both NRZ and CSB methods. *P*-value distributions were compared using Q-Q plots (columns 1 and 2) and scatter plots of *log* (*p*) values (column 3). Q-Q plots in column 1 display both the total distributions (black lines), i.e. regardless of GS size, and distribution fractions that correspond to smaller GS (*N*_AGS_ + *N*_FGS_, color lines). The QQ-plots in column 2 are insets of the black, un-stratified Q-Q plots of column 1. Identity lines (x = y) are plotted in dotted grey and dotted red in the QQ-plots and scatter plots, respectively. Analogous plots in regard of other factors biasing NRZ *p*-values (*C*_AGS_ + *C*_FGS_ and *N*_edges_) are provided in Additional file 1: Figure S1 (note that plot columns 2 and 3 are the same as in the present figure)

We compared the *p*-value distributions between the two methods (Fig. 2). The both were capable of adjusting *p*-values for multiple testing, but we omit the adjusted value analysis here since our GS test set of 330 FGS (mostly KEGG pathways with addition of GO terms and other sets related to cancer, cytokine signaling, inter-cellular communications etc. [10] abounded with highly functionally similar GS pairs and thus the fraction of significant q-values was ~30%. We instead focus on the *p*-values in order not to miss important distribution details. It is apparent that the estimates from network randomization become more consistent with the growing number of randomization runs, from *N* = 3 to *N* = 300. The third column of Fig. 2 displays strong and asymptotically increasing correlation between *p*-values from the network randomization z-test (NRZ) and chi-squared binomial formula (CSB) (Spearman *r* = {0.83; 0.94; 0.97; 0.98; 0.99} for *N* = {3; 10; 30; 100; 300}, respectively). Since CSB employed a deterministic procedure, we assume that all dispersion of *p*-value points in the NRZ-CSB space should be attributed to sampling errors by the stochastic NRZ algorithm. CSB and NRZ *p*-values converged sufficiently well only at *N* > 30. According to the quantile analysis with Q-Q plots (2nd column), CSB *p*-values were more conservative than those of NRZ. Next, the latter was somewhat less sensitive than CSB in regard of small GS, especially of those where both GSs were small, below 30 or 10 nodes altogether (green and blue lines in the first column). Again, NRZ *p*-values from the computationally feasible but insufficient *N* = {3;10; 30} exhibited more disagreement with respective CSB values. In addition to the sums *N*_AGS_ + *N*_FGS_, we stratified the Q-Q plots by the sum of node degrees *C*_AGS_ + *C*_FGS_ and by the actual number of edges between two GSs, *N*_edges_ (Additional file 1: Figure S1A and B). The sum *C*_AGS_ + *C*_FGS_ appeared a strongly biasing factor (although *N* and *C* expectedly correlated in the test set). The influence of *N*_edges_ was almost non-existent.

Since there was no gold standard for network enrichment, we could not unambiguously conclude that CSB was more precise than NRZ. However it seems to be preferable because of its, in general, more conservative estimates and, in particular, higher sensitivity on small GS as well as the convergence of NRZ at higher values of N. The NRZ-specific bias on small GSs likely occurred because between-GS connectivity values could not take on negative values, hence the left tail of the distribution of *N*_edges_ was compressed and standard deviation must be underestimated.

Thus while using NRZ, the deviation from CSB values and bias due to small GS size were considerably large at *N* < 100. However, performing many randomizations might make the procedure prohibitively slow. While using NRZ, the computation time grows linearly with the number of randomizations. For example on a server running 2.6.32-642.el6.x86_64 (OS Scientific Linux),one randomization of the given network took around 3 min plus 20 min for counting connectivity in the given FGS collection, so that the total time for 100 randomizations was 100*(20 + 3) = 2300 min. For the most direct comparison, the same perl software [9] in the CSB mode, i.e. without network randomization, run 15 min. In the both cases, the perl process occupied between 300 and 400 MB RAM. Running R package NEArender required less than 5 min and below 200 MB RAM. We note that in many practical tasks the number of either AGS or FGS could be significantly reduced. However as stated above, the current package is mostly meant for generating NEA scores that could be used as predictive features in machine learning - and those AGSxFGS matrices are supposed to be large.

### Robustness of results obtained from different replicates

Statistically underpowered experimental designs are not welcomed in the scientific community but, due to the lack of resources, are still practiced. In particular, it is common to represent patients in large cohort screens with single samples [11, 12]. Both the GSEA and NEA analyses measure enrichment in order to summarize signals from individual genes' to the level of pathways and biological processes. This feature suggests a potential increase in robustness of conclusions in experiments that lack replicates. We decided to investigate this robustness of using single-gene expression values versus GSEA and NEA under different scenarios using cell transcriptome data from the FANTOM5 CAGE RNA sequencing set [13]. In order to make results comparable between NEA and GSEA, we use the binomial version of the latter [1], which allowed applying the analyses to the same input gene sets. Commonly, statistical power is analyzed via extrapolating variance estimates [14, 15]. Since GSEA and NEA do not provide such estimates, we instead watched consistency of conclusions drawn from individual replicates.

The samples, in 3 to 5 biological (distinct healthy individuals) replicates, originated from different fibroblast, epithelial, and smooth muscle cells. In total, 43 replicates from 11 cell types quantified RNA transcript tags mapped to 16620 genes. This provided a broad range of degrees of dissimilarity between type-specific transcriptomes. The dissimilarity was evaluated as a fraction of significantly DE genes in fully replicated designs and ranged from 2.4% (“fibroblast.periodontal” vs. “fibroblast.gingivial”) to 54.8% (“smooth.muscle.umbilical.artery” vs. “gingival.epithelial”) DE genes. This set-up allowed us to model situations of analyzing DE of each gene *g* using single samples, e.g. *g*_*Ai*_ vs. *g*_*Bj*_ on samples *i* and *j* from cell types *A* and *B*, and then using all available replicates for *A* and *B* (*N*_*A*_, *N*_*B*_): ${g}_{A\left\{1\dots {N}_A\right\}}$ vs. ${g}_{B\left\{1\dots {N}_B\right\}}$. Note that calculating DE *p*-values was thus possible only in the latter case. Otherwise, DE could only be ranked by values of fold change between *A*_*i*_ and *B*_*j*_. We quantified DE in all the 55 possible *A*_*i*_ - *B*_*j*_ pairs between the 11 cell types. As an example, if there were *N*_*A*_ = 3 and *N*_*B*_ = 5 replicates, then we could model DE measurements on 5*3 = 15 pairs of single samples plus one analysis ${g}_{A\left\{1\dots {N}_A\right\}}$ vs. ${g}_{B\left\{1\dots {N}_B\right\}}$. In the following, we review preservation of DE estimates across different cell samples contrasts analyzed with three different methods: the default DE analysis on individual genes, GSEA, and NEA. The preservation was evaluated in the form of Spearman rank correlation coefficients between: 1) fold change values of individual genes for gene-wise analysis“as is”,2) *P*-values of FGS enrichment scores obtained with binomial GSEA, and 3) *P*-values of FGS enrichment scores obtained with CSB NEA.

**Fig. 3:**
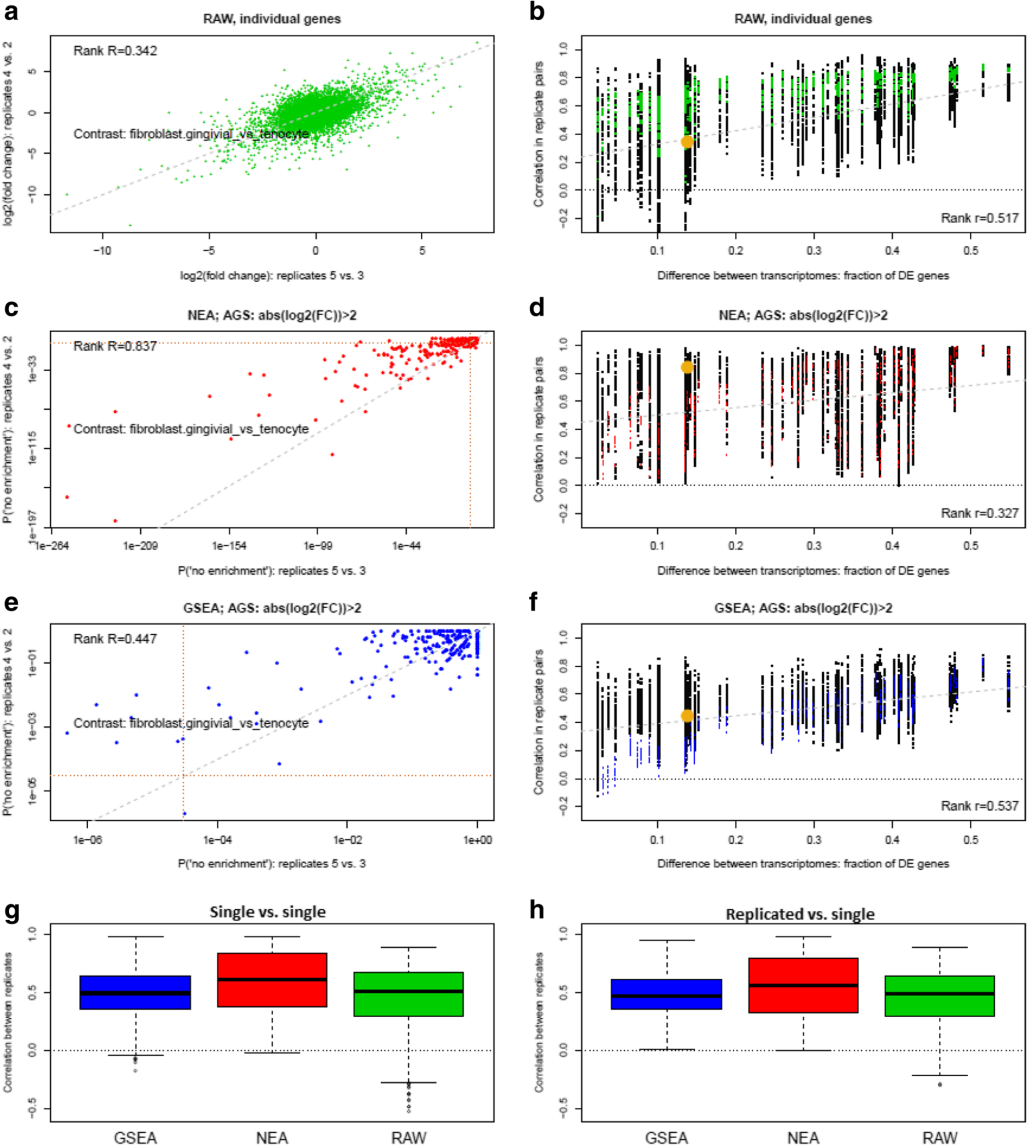
Agreement between biological replicates in alternative approaches to differential expression analysis. **a**, **c**, **e**: Examples of Spearman rank correlations between DE analyses without replicates on gingivial epithelial versus tenocyte cells using samples from two different donor pairs (gingivial epithelial: #4 and #5; tenocyte: #2 and #3). Since *p*-values in the non-replicated DE analyses were not available, the plot A (RAW) represents raw fold change values for 16620 genes, while plots C and E represent *p*-values for 330 FGSs. In C and E, the vertical and horizontal brown dotted lines delineate *p*-values significant after the Bonferroni correction. Dashed grey line: the linear regression fit of Y on X (the R values shown in the corners were calculated using the rank formula and are thus independent of the fits). NEA and GSEA values were obtained for AGSs representing DE genes in each analysis that satisfied the criterion abs (log _2_ (fold change))>2, i.e. the 4-fold change in either direction. These example rank R values from A, C, and E are plotted at respectively B, D, and F as big orange dots. Thus in B, D, and F, the Spearman coefficients from A, C, and E (top left corner) as well as from all other pairwise comparisons are plotted as a function of relative difference strength between respective transcriptomes (X axis). As an example, according to the results of fully powered DE analysis for plots A, C, and E, 13.8% genes were DE with adjusted *p*-value < 0.05. Hence the value 0.138 is used as X-coordinate for the orange dots. The grey dotted linear regression line and the Spearman rank R value quantify the relations between X and Y. Black and colored points correspond to 1) correlation values of non-replicated DE analyses with each other and 2) correlations where one of the two analyses was replicated, respectively. The boxplots G and H summarize results across all cell types and AGS versions for non-replicated vs. half-replicated options (1) and (2). All pairwise contrasts of GSEA and RAW against NEA were significant with p(H0) < 0.001. In Additional file 1: Figure S3, six plots for NEA and GSEA represent results of using all the six alternative AGS versions

The example scatterplots at Fig. 3 (a, c, and e) display agreement between the same replicate pairs of the same contrast between fibroblast gingivial vs. tenocyte cells. The fold change values and *p*-values, although being different in their nature and scale, allowed us to rank the results in the same way as a biological researcher would prioritize them. We note here that in GSEA and NEA neither *p*-values nor enrichment scores (being mutually rank-invariant) explicitly provide sampling errors similar to between-sample variance in the DE analysis. All such comparisons between sample pairs were quantified by Spearman rank correlations and used as individual points in the plots on the right (b, d, and f). One observation from the plots C, and E was that regardless of the correlation strength, NEA possessed a much higher statistical power to detect enrichment – which has been discussed in details by Alexeyenko et al. in 2012 [2]. Indeed, no GSEA *p*-values appeared significant after the Bonferroni adjustment for multiple testing in either replicate pair (brown dotted vertical and horizontal lines). For comparison, almost 1/3rd of the NEA scores were significant in the both replicate pairs.

GSEA and NEA were each tested using six alternative variants of AGS, generated for each contrast: a) lists of top 100, 300, and 1000 DE genes ranked by fold change and b) lists of genes with absolute log _2_ (fold change) values exceeding 1, 2, and 4. AGS sizes of the form (b) were not fixed and depended on the magnitude of the difference between the two transcriptomes. When using all available replicates in the analyses (a), the genes were ranked by *p*-value with functions lmFit and eBayes in R package limma [16]. In (b) we required that, in addition to the fold change cutoff, the adjusted *p*-values did not exceed 0.05.

Overall, the scores from NEA agreed with each other much better than those from GSEA (Fig. 3, g and h). By investigating the AGS- and method-specific scatterplots for AGSs of type”abs (log _2_ (fold change)) > 2” (Fig. 3, b, d, and f; respective plots for all the six AGS types are available as Additional file 1: Figure S3), we observed that the level of correlation between the analyses (Y-axis) depended on strength of the pairwise difference between cell type transcriptomes, expressed as the fraction of DE genes (X-axis). Ideally, we would observe independence of the difference strength (so that ranks fully correlate regardless of fold change, *p*-value, FDR etc.) and perfect correlation between DE results from both individual sample pairs and replicated analyses. However, it was unrealistic to expect large and efficient AGSs from few (e.g. 5…7%) significantly DE genes. Still, we can conclude that NEA output was considerably closer to the desirable pattern than those of the gene-wise and GSEA analyses. Indeed, in addition to the stronger overall correlation, NEA performed particularly well on weakly different cell types pairs (see the regression lines’ intercepts with Y-axes and the rank *r* values in the bottom right corners in Fig. 3, b, d, and f). The only exception could be found for AGSs top100 (Additional file 1: Figure S3), where GSEA appeared superior over NEA. However, this option would be practically unusable in GSEA because of its low statistical power on small gene sets and, hence, few significant enrichment values.

We also could compare consistency of results obtained via i) individual sample pairs against each other (“*g*_*Ai*_ vs. *g*_*Bj*_”against “*g*_*Am*_ vs. *g*_*Bn*_”) and ii) results from individual sample pairs against the replicated analysis (“*g*_*Ai*_ vs. *g*_*Bj*_”against “${g}_{A\left\{1\dots {N}_A\right\}}$ vs. ${g}_{B\left\{1\dots {N}_B\right\}}$”). One can also see that the option (ii), i.e. the comparison of non-replicated to replicated analyses (colored points), often exhibited poorer correlation than comparisons (i) (black points), especially for GSEA (the same pattern can be seen in the boxplots of Additional file 1: Figure S2).

## Discussion

We demonstrated that estimates of enrichment from the network randomization procedure were biased. They could sufficiently converge to respective CSB values only at very large, impractical numbers of randomizations runs.

While considering the “lack-of-replicates” scenario, we assumed that the former shall always be preferred. However, our analysis demonstrated the higher (although still imperfect) robustness of NEA scores in the full absence or shortage of replicates. This conclusion about superiority of NEA over gene-wise analyses is, of course, only relevant when the research problem can be approached with an exploratory analysis at a pathway level and not when it requires identifying individual consistently deregulated genes. In addition, this advantage of NEA explains the earlier observed higher robustness of its scores, compared to individual gene profiles, as descriptors of experimental models of cancer-inhibitory fibroblasts [10]. Importantly, biological replicates of patients’ samples are rarely available in clinical cohorts, so that we anticipate efficient usage of NEA in such setups.

NEA can also be performed using the online tool available at https://www.evinet.org. The latter is more interactive, user-friendly, and focused on visualization, while the package NEArender enables high performance, possibly with parallel computation. In combination with the ancillary functions for input preparation and benchmarking NEArender is meant to become a practically useful part of bioinformatics and biostatistics R pipelines.

## Conclusions

Output of NEArender allows using sample- or patient-specific pathway scores as predictive features of phenotype or clinical variables. Compared to GSEA, this raises statistical power and robustness to practically acceptable levels. Compared to previous implementations of NEA, the analysis runs much faster and removes bias due to smaller gene set size. Package NEArender also contains previously not available ancillary functions for preparing and benchmarking input.

## Methods

### Significance estimation

NEA considers topological properties of the network via node degrees of GS members as well as the total number of edges in the whole network. For a long time, confidence of network enrichment scores has been mostly computed using network randomizations in order to estimate the connectivity value (i.e. number of edges between two GSs expected by chance) as well as its standard deviation [2, 3, 17]. Then the network enrichment statistic was computed as a z-score:

$$ z=\frac{n_{AGS- FGS}-{\widehat{n}}_{AGS- FGS}}{\sigma_{AGS- FGS}} $$

where *n*_AGS-FGS_ was the actual number of edges that connect any nodes of AGS and any nodes of FGS, while ${\widehat{n}}_{AGS- FGS}$ and *σ*_*AGS* − *FGS*_ were the average number of AGS-FGS edges and respective standard deviation found in a number of randomized instances of the actual network.

The randomization algorithm by Maslov and Sneppen [17] was based on rewiring each original edge between nodes *i and j*, so that *i* and *j* instead get connected to randomly sampled (without replacement) nodes *k* and *l*. This allowed preserving individual node degrees (i.e. the total number of edges for a given node) as well as the global topological properties of the network (first of all the scale-freeness). However, McCormack et al. [3] demonstrated that in this randomization procedure higher-order topological properties, such as the propensity of high-degree nodes to avoid connections with other high-degree nodes, could still be biased. They suggested another algorithm for cases of relevance. In our view, the removal of higher order topological biases is not always justified and the decision to apply this over-randomization should depend on a particular research question.

The focus of our present work is on creating a software implementation which is fast and independent of GS sizes and numbers of edges connecting them. In order to do that, we evaluate enrichment of AGS versus FGS using the binomial formula:

$$ {\upchi}^2=\frac{{\left({n}_{\mathrm{AGS}-\mathrm{FGS}}-{\widehat{n}}_{\mathrm{AGS}-\mathrm{FGS}}\right)}^2}{n_{\mathrm{AGS}-\mathrm{FGS}}}+\frac{{\left(!{n}_{\mathrm{AGS}-\mathrm{FGS}}-!{\widehat{n}}_{\mathrm{AGS}-\mathrm{FGS}}\right)}^2}{!{n}_{\mathrm{AGS}-\mathrm{FGS}}} $$,

The respective number of links expected by chance is calculated simply as:

$$ {\widehat{n}}_{AGS- FGS}=\frac{N_{AGS}\ast {N}_{FGS}}{2\ast {N}_{total}} $$,

where !*n* denotes “other than *n*”, *N*_*AGS*_ and *N*_*FGS*_ report the sums of connectivities of individual nodes (genes) in AGS and FGS, respectively, and *N*_*total*_ is the number of edges in the whole network. We note here that this simplified calculation is legitimate if only direct AGS-FGS edges are of interest (which is typically the case of NEA using sufficiently dense NETs, i.e. when in practically all AGS-FGS pairs both expected and actual connectivity is expressed with positive values) and if higher-order topology issues may be neglected.

The Gaussian *z*-scores are consecutively “reverse-engineered” by nea.render from *Χ* ^*2*^ scores via *p*-values of the latter. Since *Χ* ^*2*^, unlike of *z*, is only defined on the non-negative domain, *z* values are coerced negative in cases of depletion (as opposed to enrichment).

### Topology analysis

The package includes auxiliary functions for visual inspection of both second order biases (topology2nd) and scale-freeness (connectivity). The vignette to the package provides examples of various deviations found in nine example networks of different provenance.

### Parallel computation

At the most computationally intense step, which is the counting of actual network edges in each AGS-FGS pair, the package can employ parallel jobs enabled with R package parallel (https://stat.ethz.ch/R-manual/R-devel/library/parallel/doc/parallel.pdf).

### Gene set enrichment analysis

Either together with or instead of NEA, users can also perform the conventional binomial enrichment analysis GSEA (note that here the binomial analysis is applied to the gene quantities rather than to network edge counts as in NEA). It accepts the same input as nea.render (excluding NET), and produces similarly arranged output from Fisher’s exact test: odds ratio estimate, *p*-value, q-value, and the number of genes shared by AGS and FGS.

### Included example datasets

In comparison to GSEA, NEA requires an extra component: a global network of functional coupling between genes and/or proteins. The package contains the following data sets: a small and a large version of NET net.kegg [18] and net.merged [9], a collection of FGSs can.sig.go (2406 distinct genes in 34 KEGG pathways [18] and GO terms [19] as well as three inputs for creating AGSs: somatic point mutations tcga.gbm [20] and two subsets of FANTOM5 transcriptomics data fantom5.43samples and fant.carc [13].

## Additional files

Additional file 1: Figure S1. Sensitivity and sources of bias in randomization-based versus binomial calculation of network enrichment. **Figure S2.** Rank correlation coefficients between results of differential expression analysis on different sample pairs and groups (Additional file 2). **Figure S3**. Agreement between biological replicates in alternative approaches to differential expression analysis. (DOCX 1.3 mb)

Additional file 2: Supplementary File Boxplots.Rfree.P_based.pdf (PDF 136 kb)

## Abbreviations

AGS: Altered genes set
CSB: Chi-squared binomial formula
DE: Differential gene expression
FDR: False discovery rate
FGS: Functional gene set
FN: False negative
FP: False positive
GS: Gene set
GSEA: Gene set enrichment analysis
NET: Global network of functional coupling
NRZ: Network randomization z-test
TN: True negative
TP: True positive
