# Supplementary material for: NEArender: an R package for functional interpretation of ‘omics’ data via network enrichment analysis
Source: BMC Bioinformatics. 2017 Mar 23;18(Suppl 5):118. doi: 10.1186/s12859-017-1534-y (PMC5374688; doi:10.1186/s12859-017-1534-y)

Figure S1. Sensitivity and sources of bias in randomization-based versus binomial calculation of network enrichment.

Similarly to Fig. 2, network enrichment between all vs. all 330 gene sets was analyzed with both NRZ and CSB methods. P-value distributions were compared using Q-Q plots (columns 1 and 2) and scatter plots of *log(p)* values (column 3). Q-Q plots in column 1 display both the total distributions (black lines), i.e. regardless of GS size, and distribution fractions that correspond to levels of biasing factors (colored lines). The plots are analogous to Fig. 2 except the potential biasing factors: total network connectivity C_AGS_+C_FGS_ in A and the number of edges between AGS and FGS N_edges_ in B. Columns 2 and 3 are completely identical to those in Fig. 2.

A


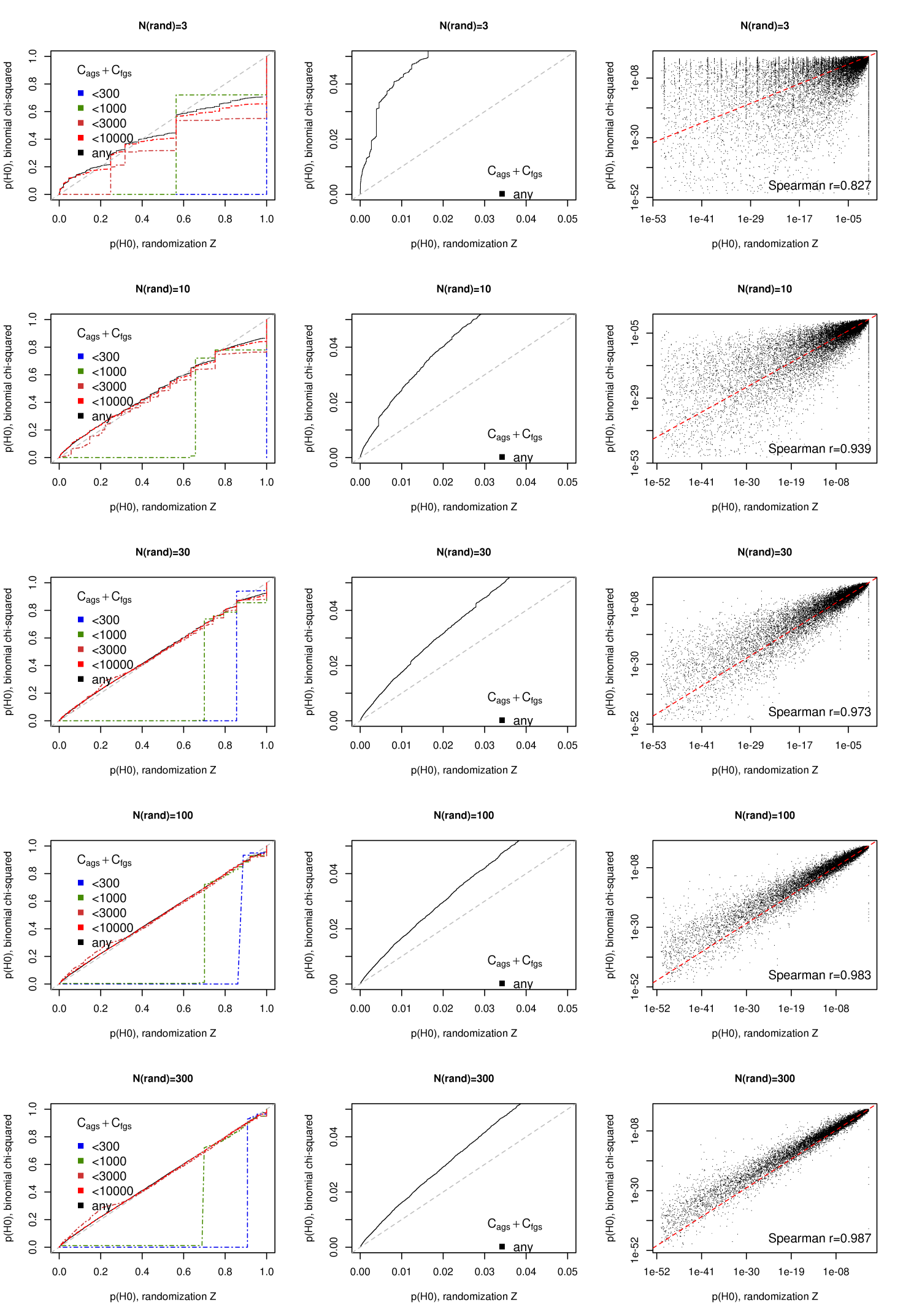


B


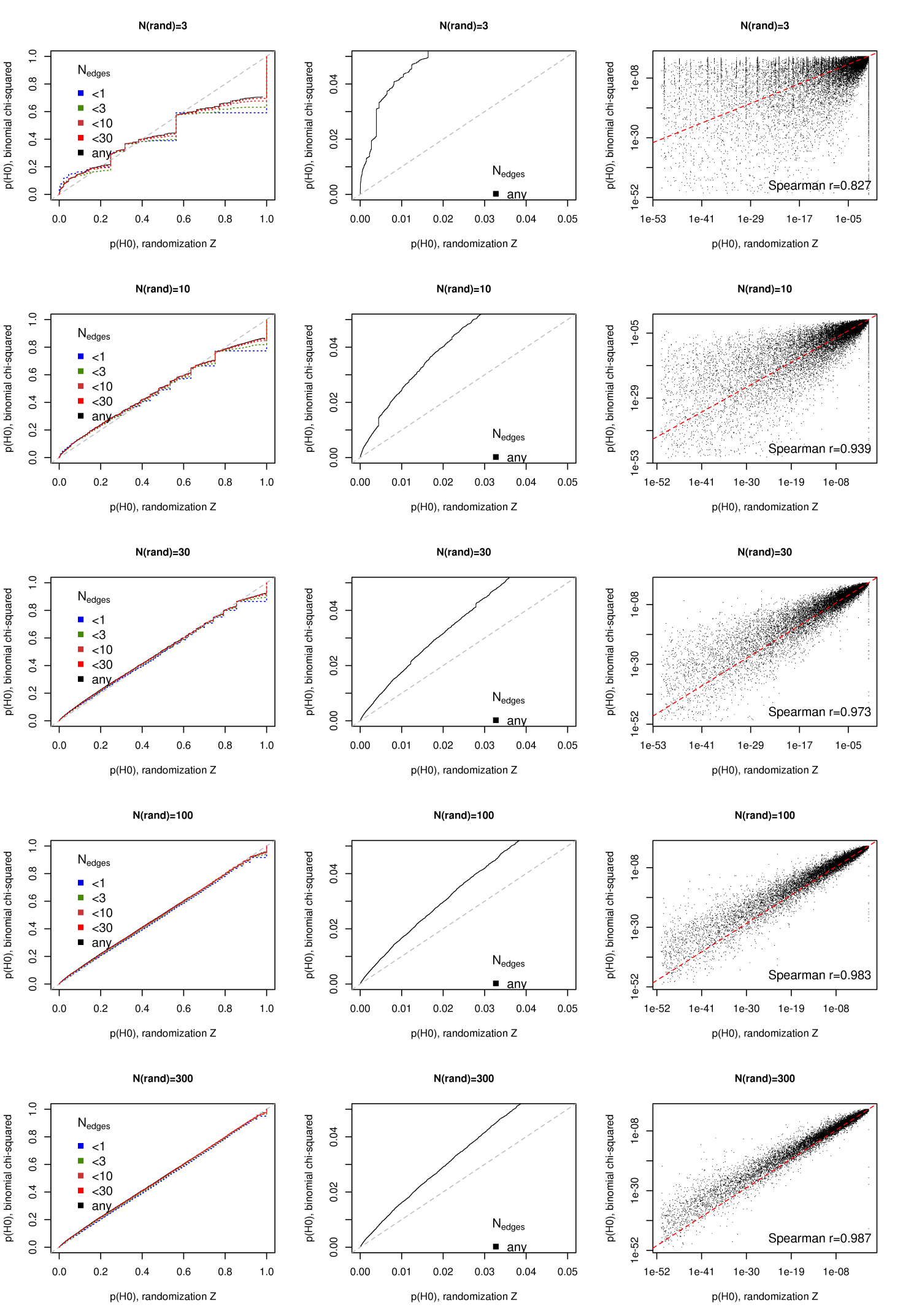


Figure S2. Rank correlation coefficients between results of differential expression analysis on different sample pairs and groups.

Each black boxplot is a summary of mutiple pairwise comparisons between single, non-replicated transcriptome samples (as exemplified at Fig. 3A).

Each colored nested boxplot is a summary of pairwise comparisons between single samples and the respective replicated DE analysis using all available samples for the cell types (the number of samples is shown in brackets at X axis).

Widths of the colored boxes reflect strengths of transcriptomic differences between two cell types, i.e. the fractions of significantly DE genes.

Green, red, and blue labels correspond to DE analysis using raw fold change values, FGS scores from NEA and FGS scores from GSEA, respectively.

All analyzed pairs are available in Supplementary File Boxplots.Rfree.P_based.pdf (34 pages) (Additional file 2).


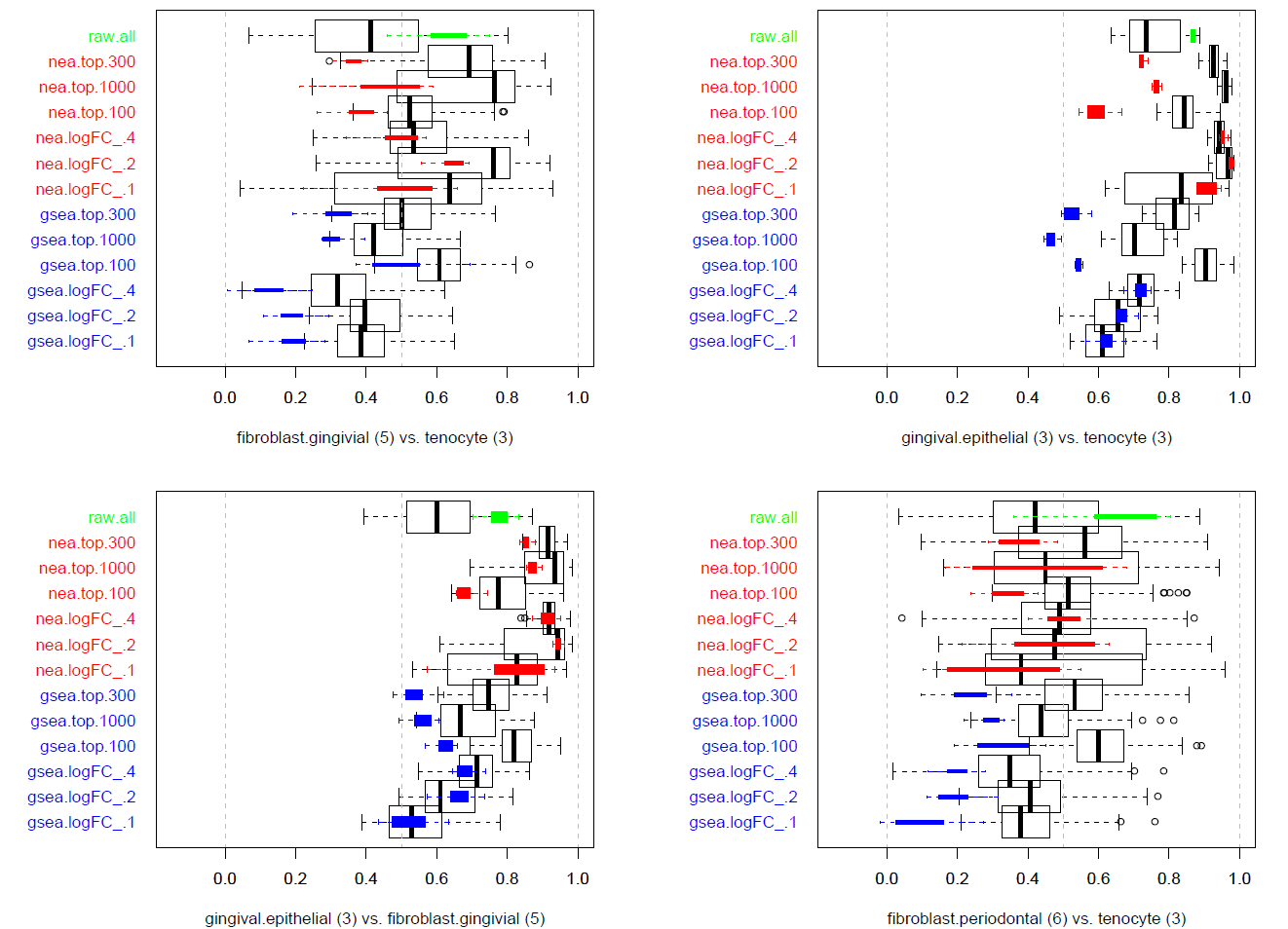


Figure S3. Agreement between biological replicates in alternative approaches to differential expression analysis.

See explanation to plots B, D, and F in the main Fig. 3.


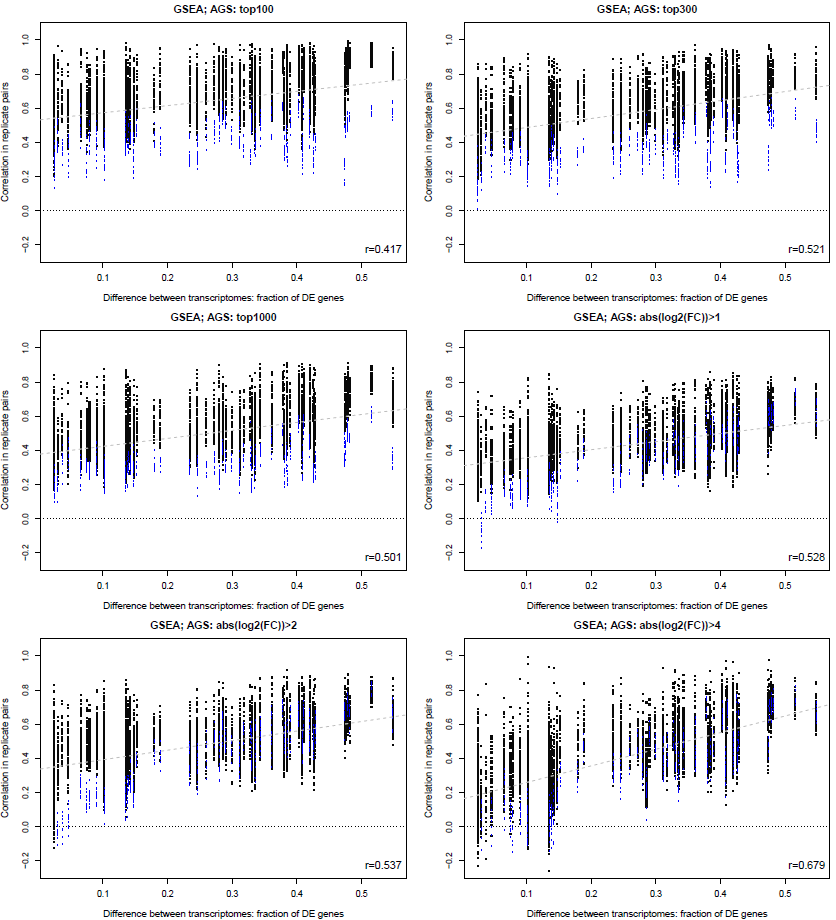

Supplement: Supplementary file 1 — Sensitivity and sources of bias in randomization-based versus binomial calculation of network enrichment. Figure S2. Rank correlation coefficients between results of differential expression analysis on different sample pairs and groups (Additional file 2). Figure S3. Agreement between biological replicates in alternative approaches to differential expression analysis. (DOCX 1.3 mb) [file 12859_2017_1534_MOESM1_ESM.docx]
