# Supplementary material for: NEArender: an R package for functional interpretation of ‘omics’ data via network enrichment analysis
Source: BMC Bioinformatics. 2017 Mar 23;18(Suppl 5):118. doi: 10.1186/s12859-017-1534-y (PMC5374688; doi:10.1186/s12859-017-1534-y)
Supplement: Supplementary file 2 — Supplementary File Boxplots.Rfree.P_based.pdf (PDF 136 kb) [file 12859_2017_1534_MOESM2_ESM.pdf]

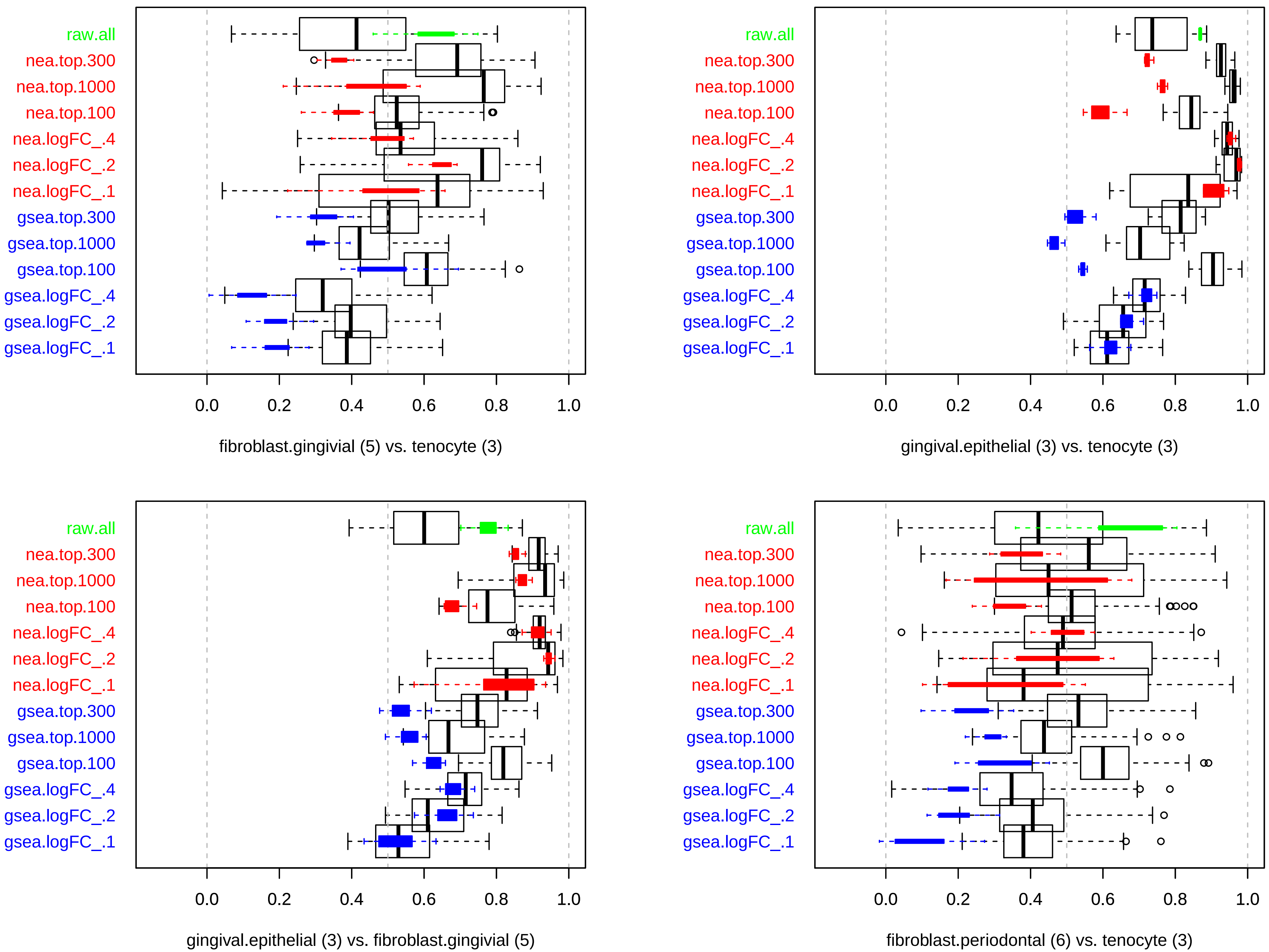
raw.all
nea.top.300
nea.top.1000
nea.top.100
nea.logFC_.4
nea.logFC_.2
nea.logFC_.1
gsea.top.300
gsea.top.1000
gsea.top.100
gsea.logFC_.4
gsea.logFC_.2
gsea.logFC_.1
0.0
0.2
0.4
0.6
0.8
1.0
fibroblast.gingivial (5) vs. tenocyte (3)
gingival.epithelial (3) vs. tenocyte (3)
gingival.epithelial (3) vs. fibroblast.gingivial (5)
fibroblast.periodontal (6) vs. tenocyte (3)

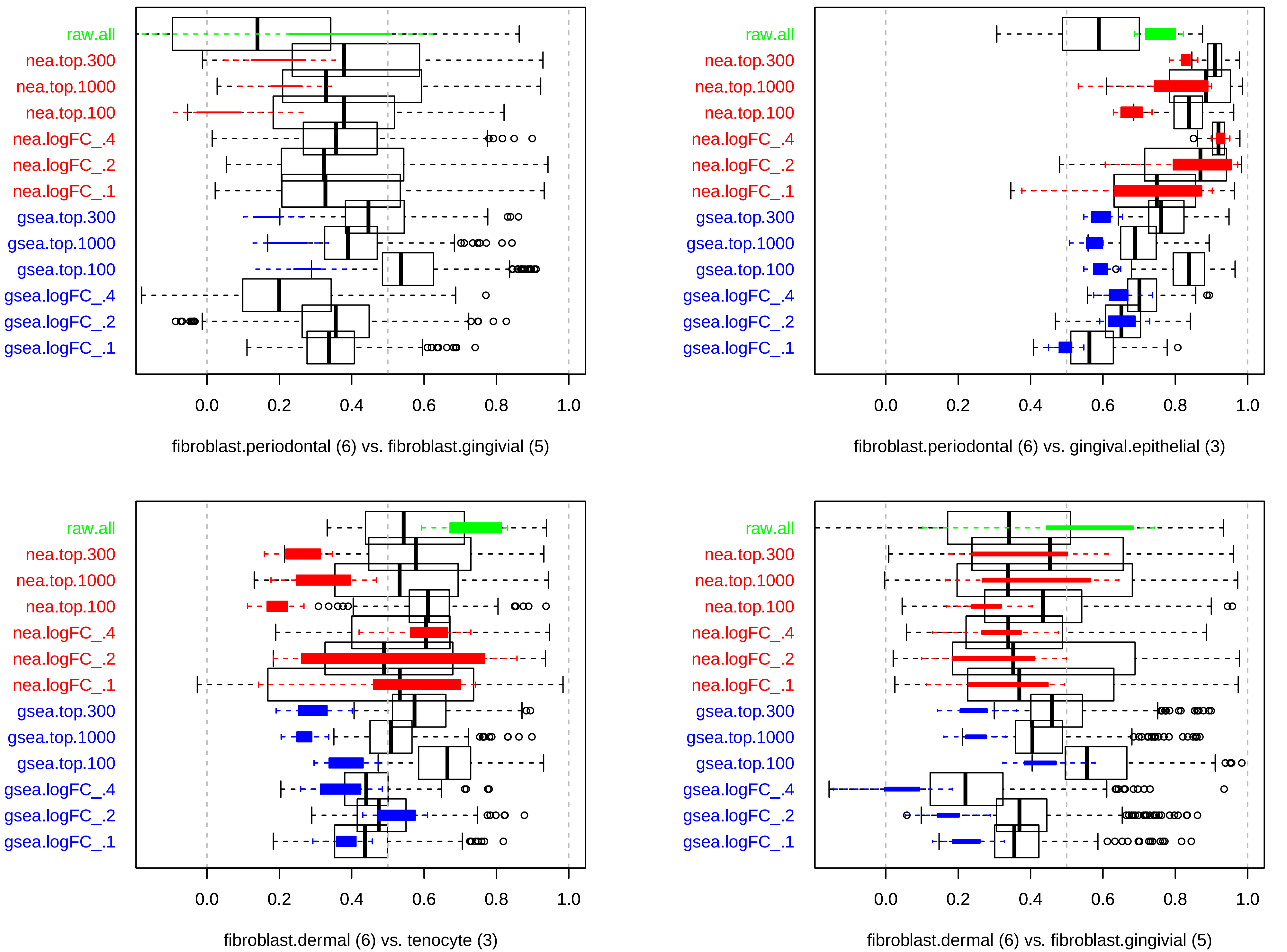

raw.all
nea.top.300
nea.top.1000
nea.top.100
nea.logFC_.4
nea.logFC_.2
nea.logFC_.1
gsea.top.300
gsea.top.1000
gsea.top.100
gsea.logFC_.4
gsea.logFC_.2
gsea.logFC_.1
0.0
0.2
0.4
0.6
0.8
1.0
fibroblast.periodontal (6) vs. fibroblast.gingivial (5)
fibroblast.periodontal (6) vs. gingival.epithelial (3)
fibroblast.dermal (6) vs. tenocyte (3)
fibroblast.dermal (6) vs. fibroblast.gingivial (5)

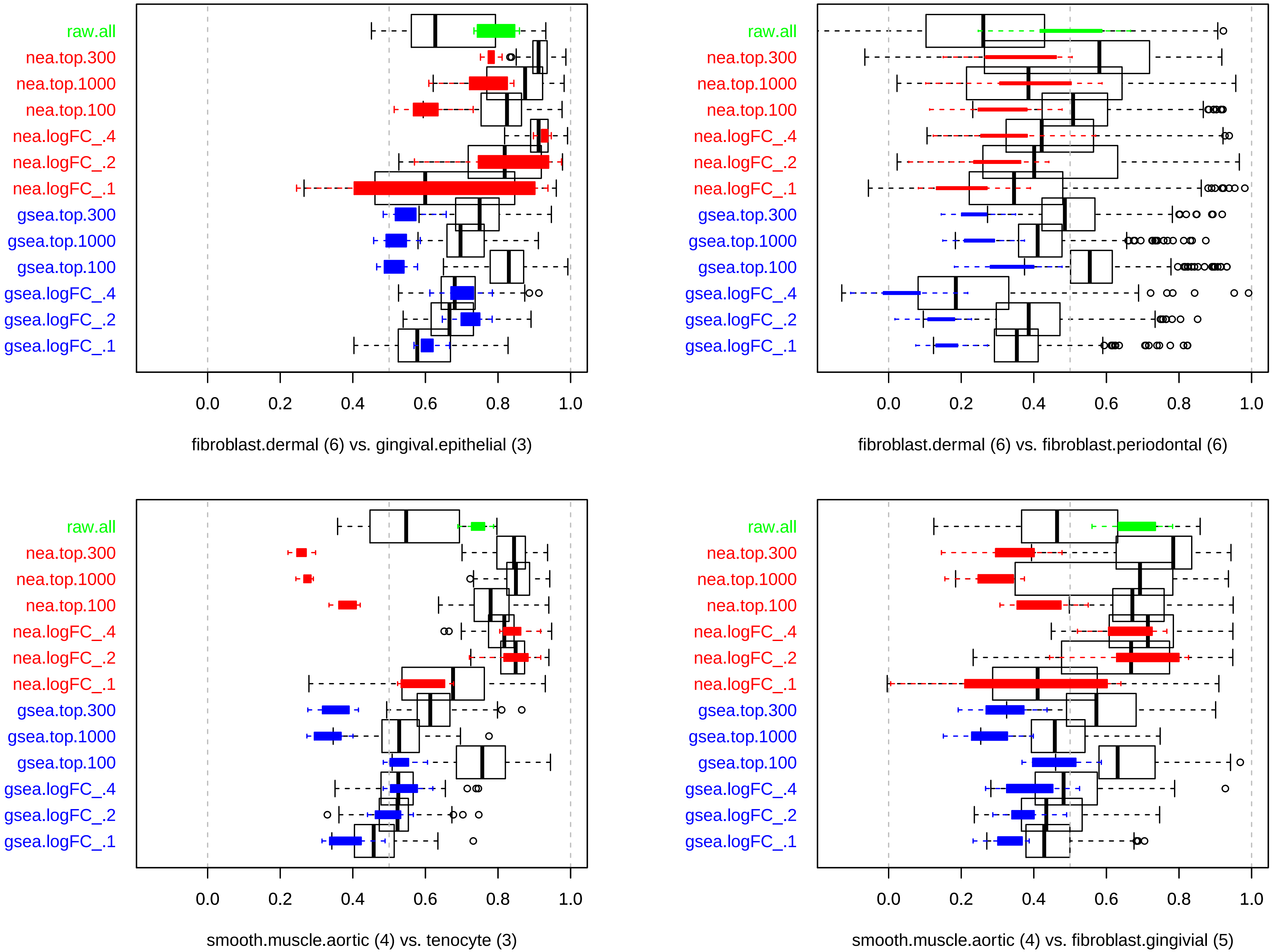
raw.all
nea.top.300
nea.top.1000
nea.top.100
nea.logFC_.4
nea.logFC_.2
nea.logFC_.1
gsea.top.300
gsea.top.1000
gsea.top.100
gsea.logFC_.4
gsea.logFC_.2
gsea.logFC_.1
0.0 0.2 0.4 0.6 0.8 1.0
fibroblast.dermal (6) vs. gingival.epithelial (3)
raw.all
nea.top.300
nea.top.1000
nea.top.100
nea.logFC_.4
nea.logFC_.2
nea.logFC_.1
gsea.top.300
gsea.top.1000
gsea.top.100
gsea.logFC_.4
gsea.logFC_.2
gsea.logFC_.1
0.0 0.2 0.4 0.6 0.8 1.0
fibroblast.dermal (6) vs. fibroblast.periodontal (6)
raw.all
nea.top.300
nea.top.1000
nea.top.100
nea.logFC_.4
nea.logFC_.2
nea.logFC_.1
gsea.top.300
gsea.top.1000
gsea.top.100
gsea.logFC_.4
gsea.logFC_.2
gsea.logFC_.1
0.0 0.2 0.4 0.6 0.8 1.0
smooth.muscle.aortic (4) vs. tenocyte (3)
raw.all
nea.top.300
nea.top.1000
nea.top.100
nea.logFC_.4
nea.logFC_.2
nea.logFC_.1
gsea.top.300
gsea.top.1000
gsea.top.100
gsea.logFC_.4
gsea.logFC_.2
gsea.logFC_.1
0.0 0.2 0.4 0.6 0.8 1.0
smooth.muscle.aortic (4) vs. fibroblast.gingivial (5)

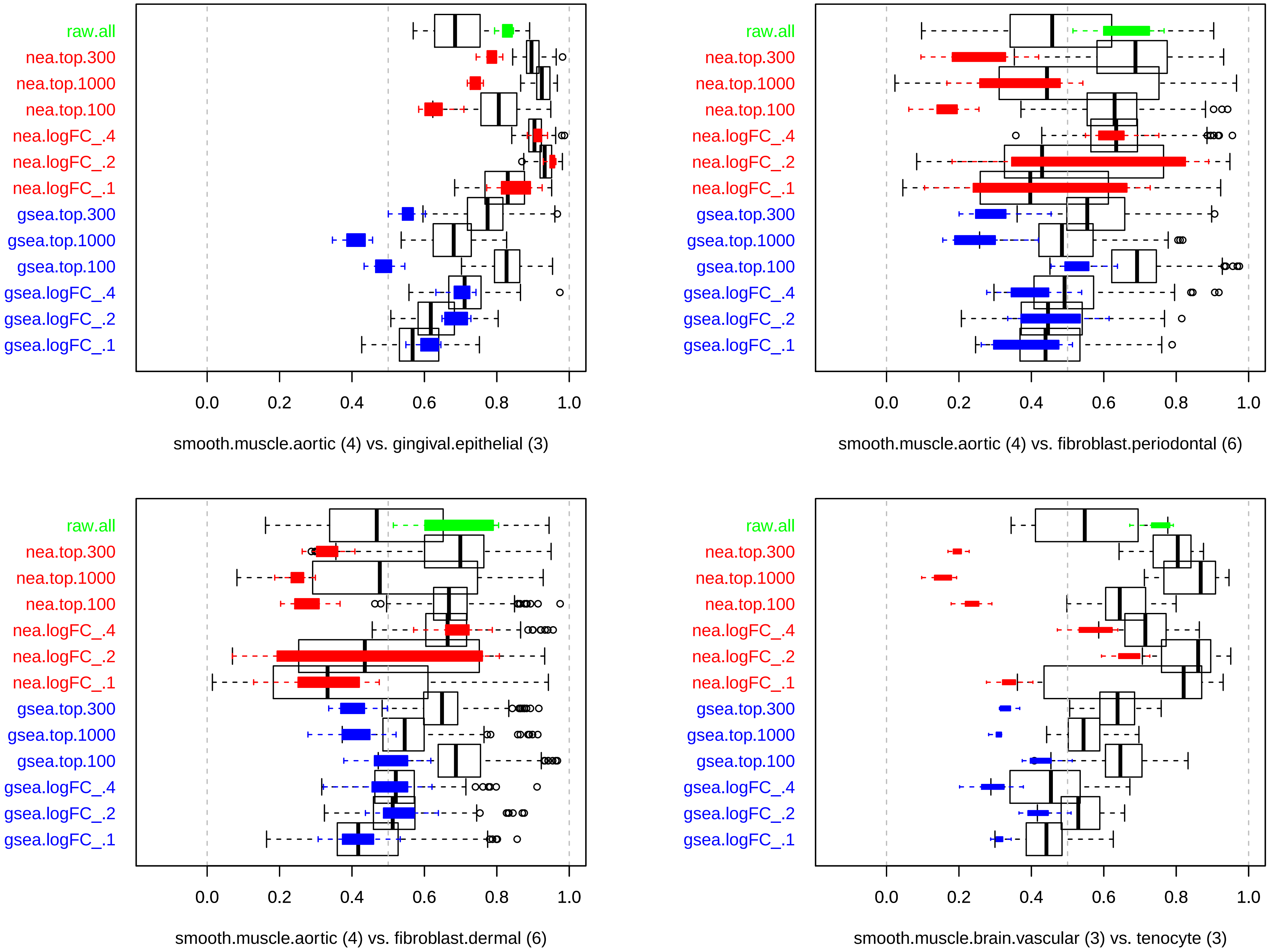
raw.all
nea.top.300
nea.top.1000
nea.top.100
nea.logFC_.4
nea.logFC_.2
nea.logFC_.1
gsea.top.300
gsea.top.1000
gsea.top.100
gsea.logFC_.4
gsea.logFC_.2
gsea.logFC_.1
0.0
0.2
0.4
0.6
0.8
1.0
smooth.muscle.aortic (4) vs. gingival.epithelial (3)
smooth.muscle.aortic (4) vs. fibroblast.periodontal (6)
smooth.muscle.aortic (4) vs. fibroblast.dermal (6)
smooth.muscle.brain.vascular (3) vs. tenocyte (3)

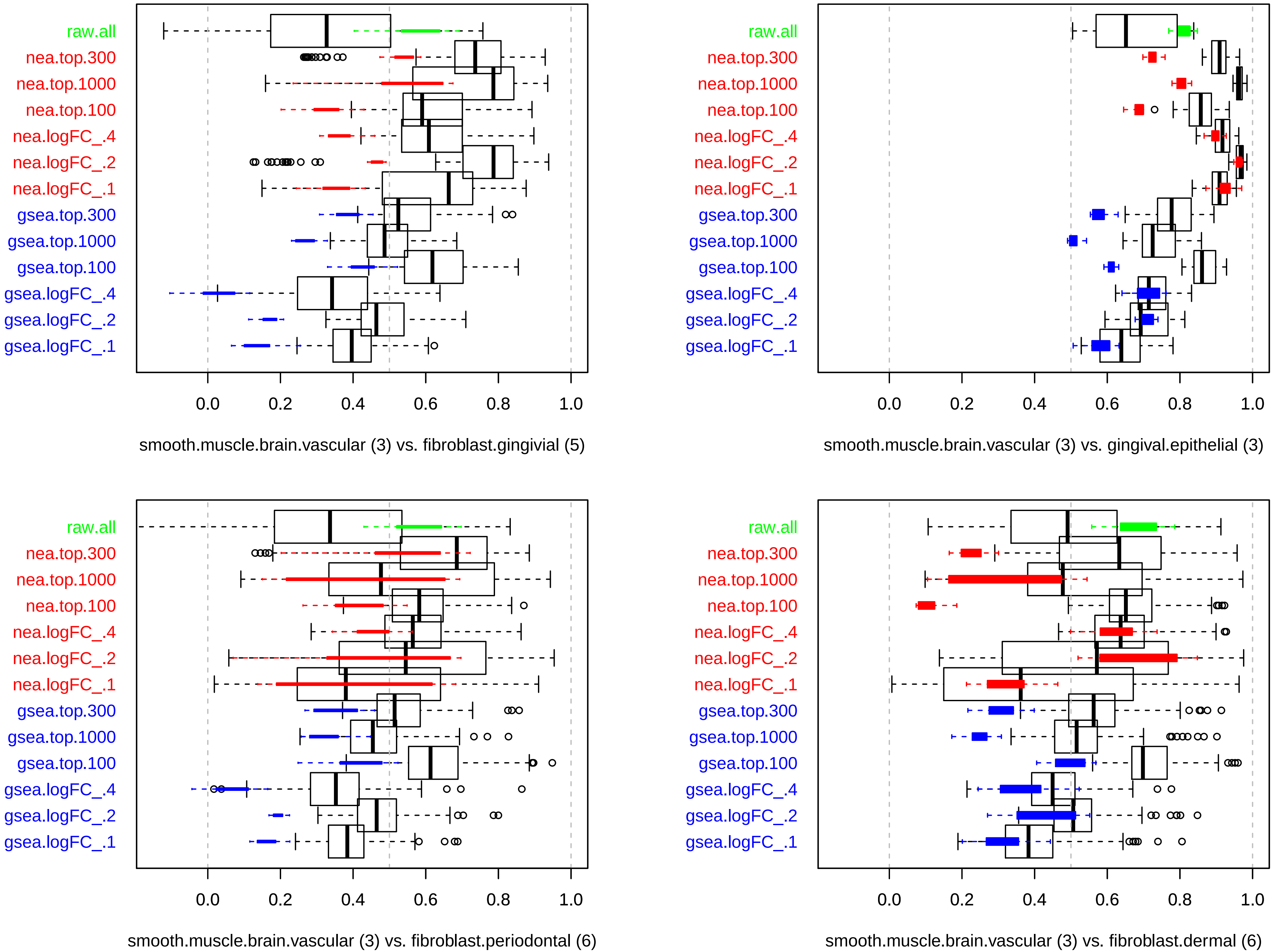
raw.all
nea.top.300
nea.top.1000
nea.top.100
nea.logFC_.4
nea.logFC_.2
nea.logFC_.1
gsea.top.300
gsea.top.1000
gsea.top.100
gsea.logFC_.4
gsea.logFC_.2
gsea.logFC_.1
0.0
0.2
0.4
0.6
0.8
1.0
smooth.muscle.brain.vascular (3) vs. fibroblast.gingivial (5)
smooth.muscle.brain.vascular (3) vs. gingival.epithelial (3)
smooth.muscle.brain.vascular (3) vs. fibroblast.periodontal (6)
smooth.muscle.brain.vascular (3) vs. fibroblast.dermal (6)

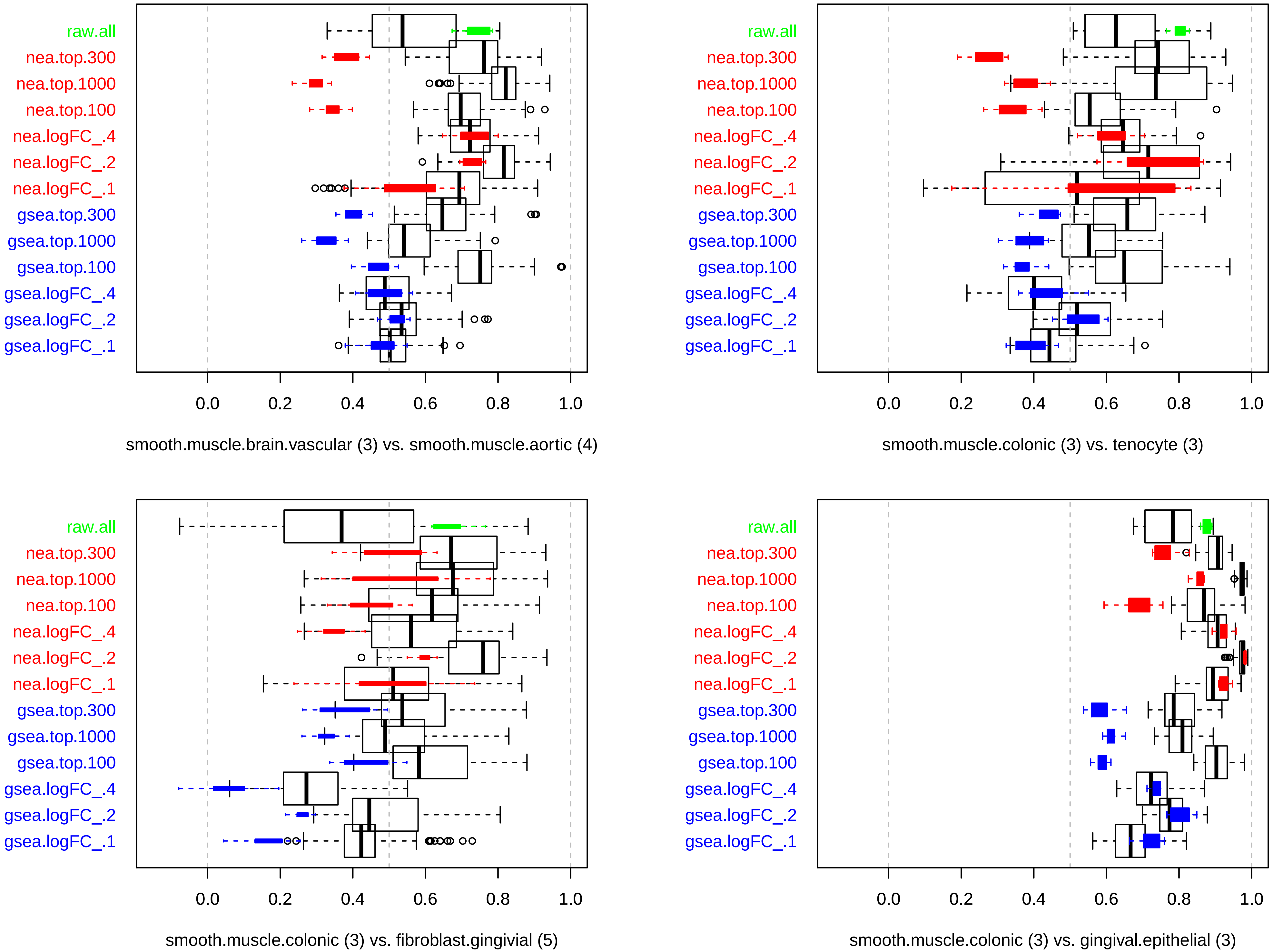
raw.all
nea.top.300
nea.top.1000
nea.top.100
nea.logFC_.4
nea.logFC_.2
nea.logFC_.1
gsea.top.300
gsea.top.1000
gsea.top.100
gsea.logFC_.4
gsea.logFC_.2
gsea.logFC_.1
0.0
0.2
0.4
0.6
0.8
1.0
smooth.muscle.brain.vascular (3) vs. smooth.muscle.aortic (4)
smooth.muscle.colonic (3) vs. tenocyte (3)
smooth.muscle.colonic (3) vs. fibroblast.gingivial (5)
smooth.muscle.colonic (3) vs. gingival.epithelial (3)

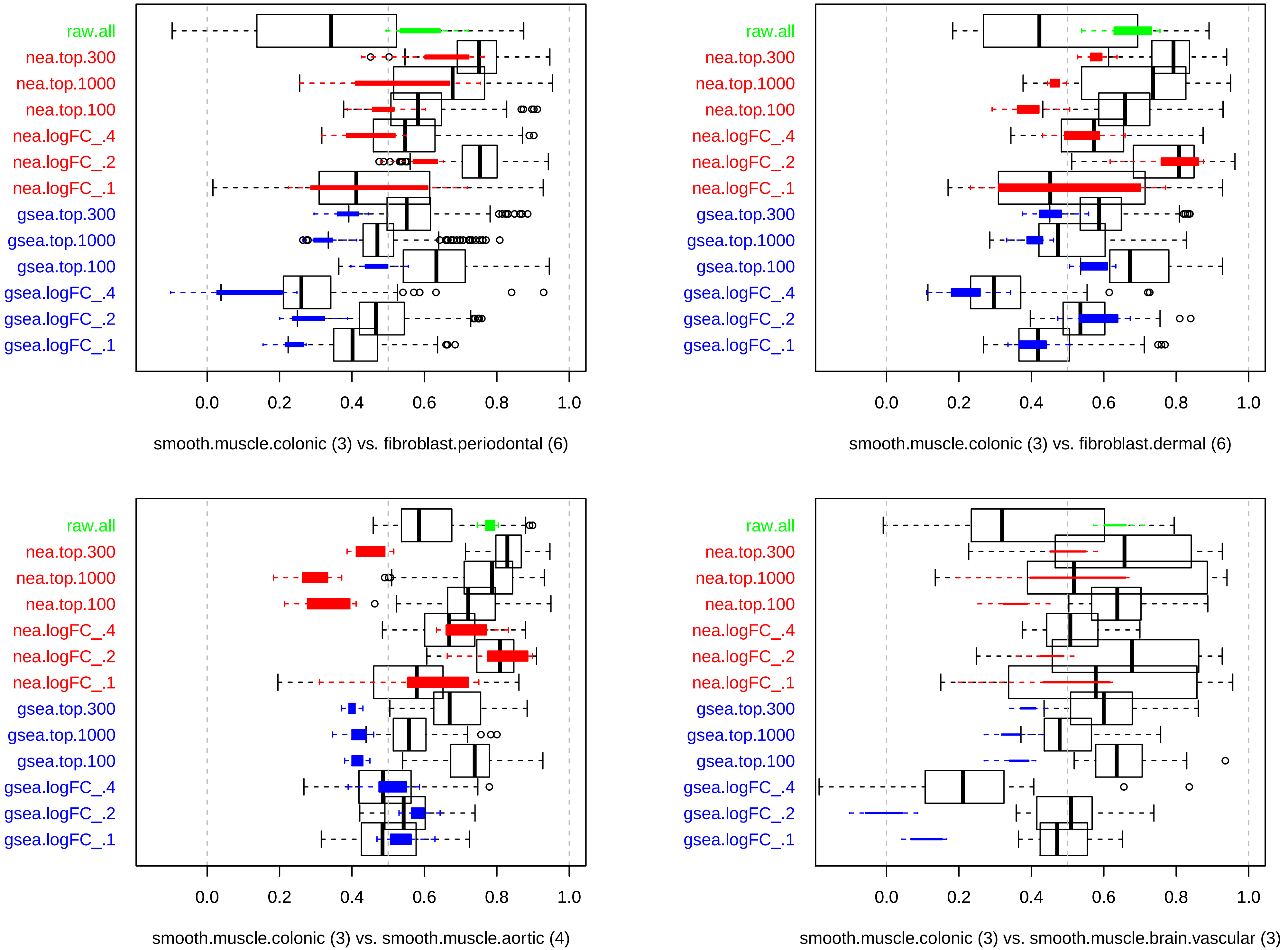

raw.all
nea.top.300
nea.top.1000
nea.top.100
nea.logFC_.4
nea.logFC_.2
nea.logFC_.1
gsea.top.300
gsea.top.1000
gsea.top.100
gsea.logFC_.4
gsea.logFC_.2
gsea.logFC_.1
0.0
0.2
0.4
0.6
0.8
1.0
smooth.muscle.colonic (3) vs. fibroblast.periodontal (6)
smooth.muscle.colonic (3) vs. fibroblast.dermal (6)
smooth.muscle.colonic (3) vs. smooth.muscle.aortic (4)
smooth.muscle.colonic (3) vs. smooth.muscle.brain.vascular (3)

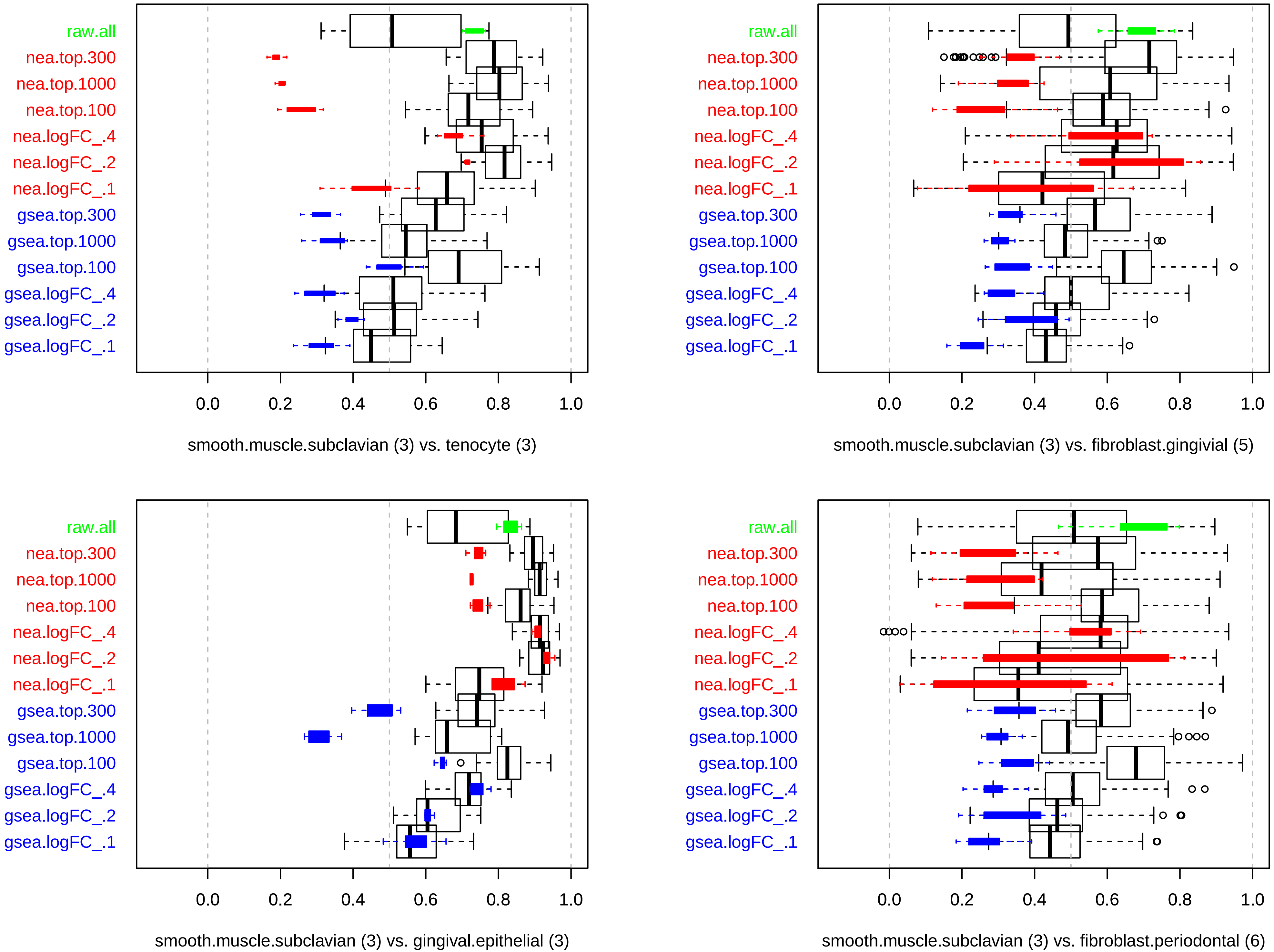
raw.all
nea.top.300
nea.top.1000
nea.top.100
nea.logFC_.4
nea.logFC_.2
nea.logFC_.1
gsea.top.300
gsea.top.1000
gsea.top.100
gsea.logFC_.4
gsea.logFC_.2
gsea.logFC_.1
0.0
0.2
0.4
0.6
0.8
1.0
smooth.muscle.subclavian (3) vs. tenocyte (3)
smooth.muscle.subclavian (3) vs. fibroblast.gingivial (5)
smooth.muscle.subclavian (3) vs. gingival.epithelial (3)
smooth.muscle.subclavian (3) vs. fibroblast.periodontal (6)

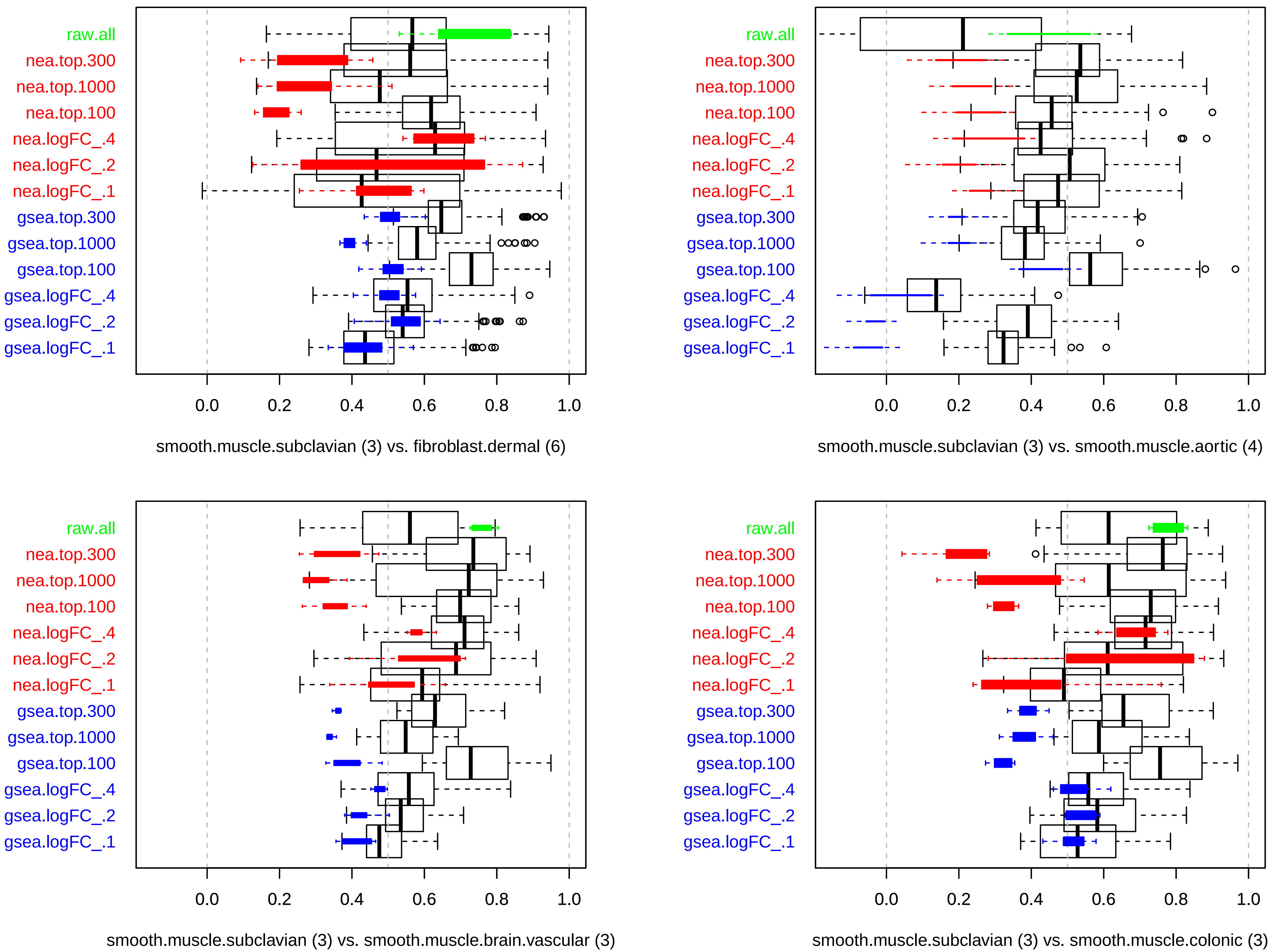
raw.all
nea.top.300
nea.top.1000
nea.top.100
nea.logFC_.4
nea.logFC_.2
nea.logFC_.1
gsea.top.300
gsea.top.1000
gsea.top.100
gsea.logFC_.4
gsea.logFC_.2
gsea.logFC_.1
0.0
0.2
0.4
0.6
0.8
1.0
smooth.muscle.subclavian (3) vs. fibroblast.dermal (6)
smooth.muscle.subclavian (3) vs. smooth.muscle.aortic (4)
smooth.muscle.subclavian (3) vs. smooth.muscle.brain.vascular (3)
smooth.muscle.subclavian (3) vs. smooth.muscle.colonic (3)

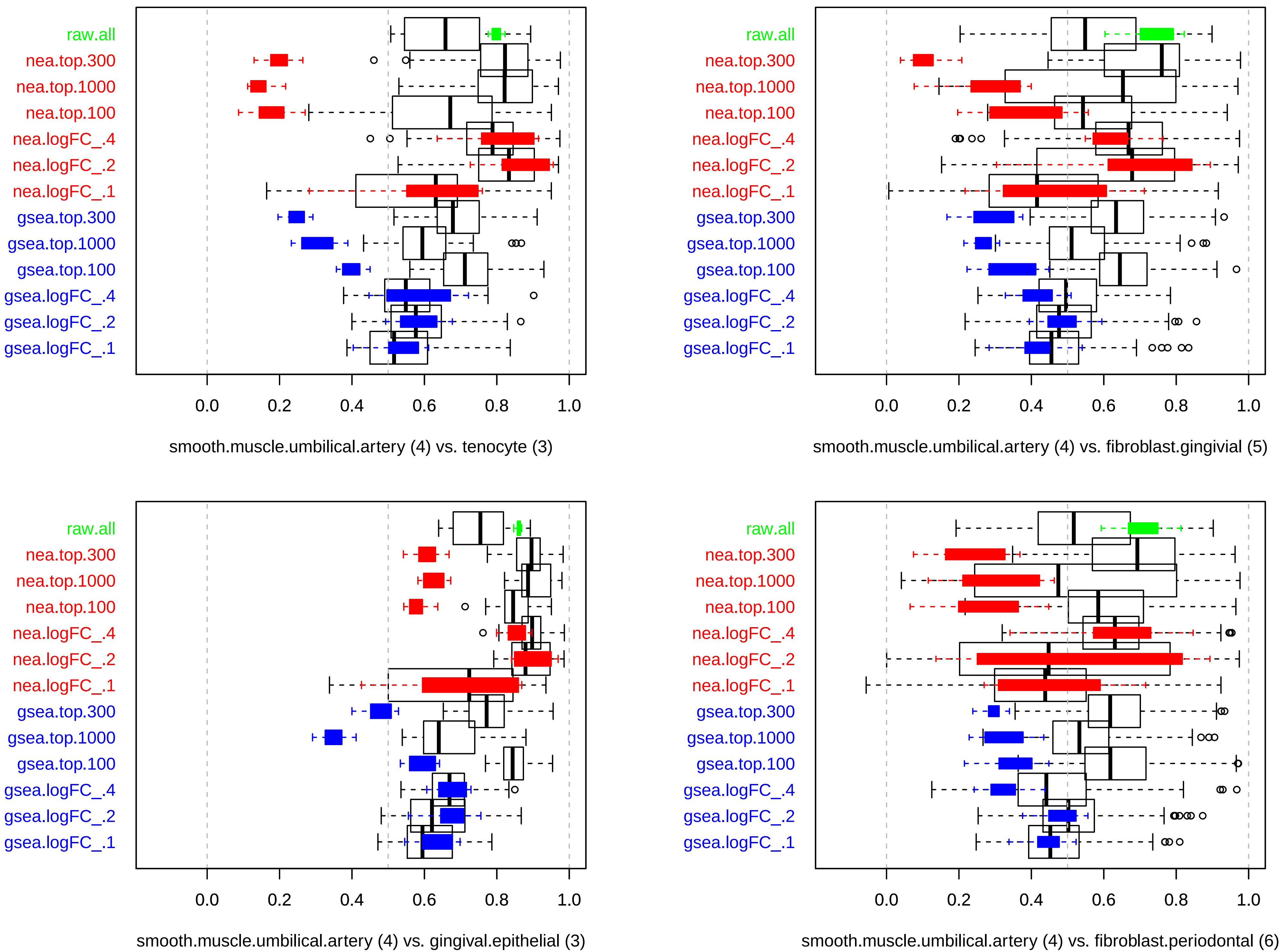

raw.all
nea.top.300
nea.top.1000
nea.top.100
nea.logFC_.4
nea.logFC_.2
nea.logFC_.1
gsea.top.300
gsea.top.1000
gsea.top.100
gsea.logFC_.4
gsea.logFC_.2
gsea.logFC_.1
0.0
0.2
0.4
0.6
0.8
1.0
smooth.muscle.umbilical.artery (4) vs. tenocyte (3)
raw.all
nea.top.300
nea.top.1000
nea.top.100
nea.logFC_.4
nea.logFC_.2
nea.logFC_.1
gsea.top.300
gsea.top.1000
gsea.top.100
gsea.logFC_.4
gsea.logFC_.2
gsea.logFC_.1
0.0
0.2
0.4
0.6
0.8
1.0
smooth.muscle.umbilical.artery (4) vs. fibroblast.gingivial (5)
raw.all
nea.top.300
nea.top.1000
nea.top.100
nea.logFC_.4
nea.logFC_.2
nea.logFC_.1
gsea.top.300
gsea.top.1000
gsea.top.100
gsea.logFC_.4
gsea.logFC_.2
gsea.logFC_.1
0.0
0.2
0.4
0.6
0.8
1.0
smooth.muscle.umbilical.artery (4) vs. gingival.epithelial (3)
raw.all
nea.top.300
nea.top.1000
nea.top.100
nea.logFC_.4
nea.logFC_.2
nea.logFC_.1
gsea.top.300
gsea.top.1000
gsea.top.100
gsea.logFC_.4
gsea.logFC_.2
gsea.logFC_.1
0.0
0.2
0.4
0.6
0.8
1.0
smooth.muscle.umbilical.artery (4) vs. fibroblast.periodontal (6)

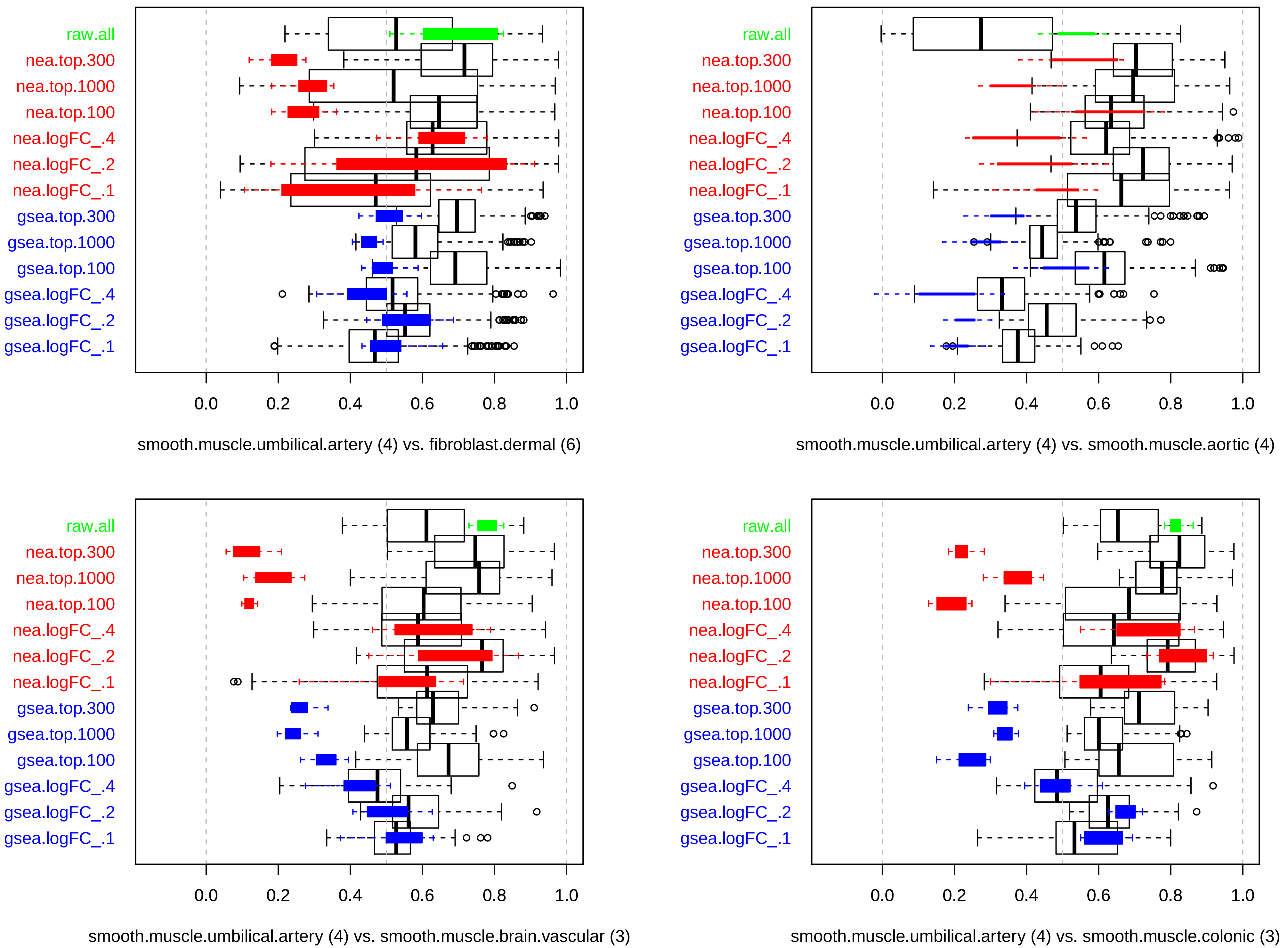

raw.all
nea.top.300
nea.top.1000
nea.top.100
nea.logFC_.4
nea.logFC_.2
nea.logFC_.1
gsea.top.300
gsea.top.1000
gsea.top.100
gsea.logFC_.4
gsea.logFC_.2
gsea.logFC_.1
0.0
0.2
0.4
0.6
0.8
1.0
smooth.muscle.umbilical.artery (4) vs. fibroblast.dermal (6)
smooth.muscle.umbilical.artery (4) vs. smooth.muscle.aortic (4)
smooth.muscle.umbilical.artery (4) vs. smooth.muscle.brain.vascular (3)
smooth.muscle.umbilical.artery (4) vs. smooth.muscle.colonic (3)

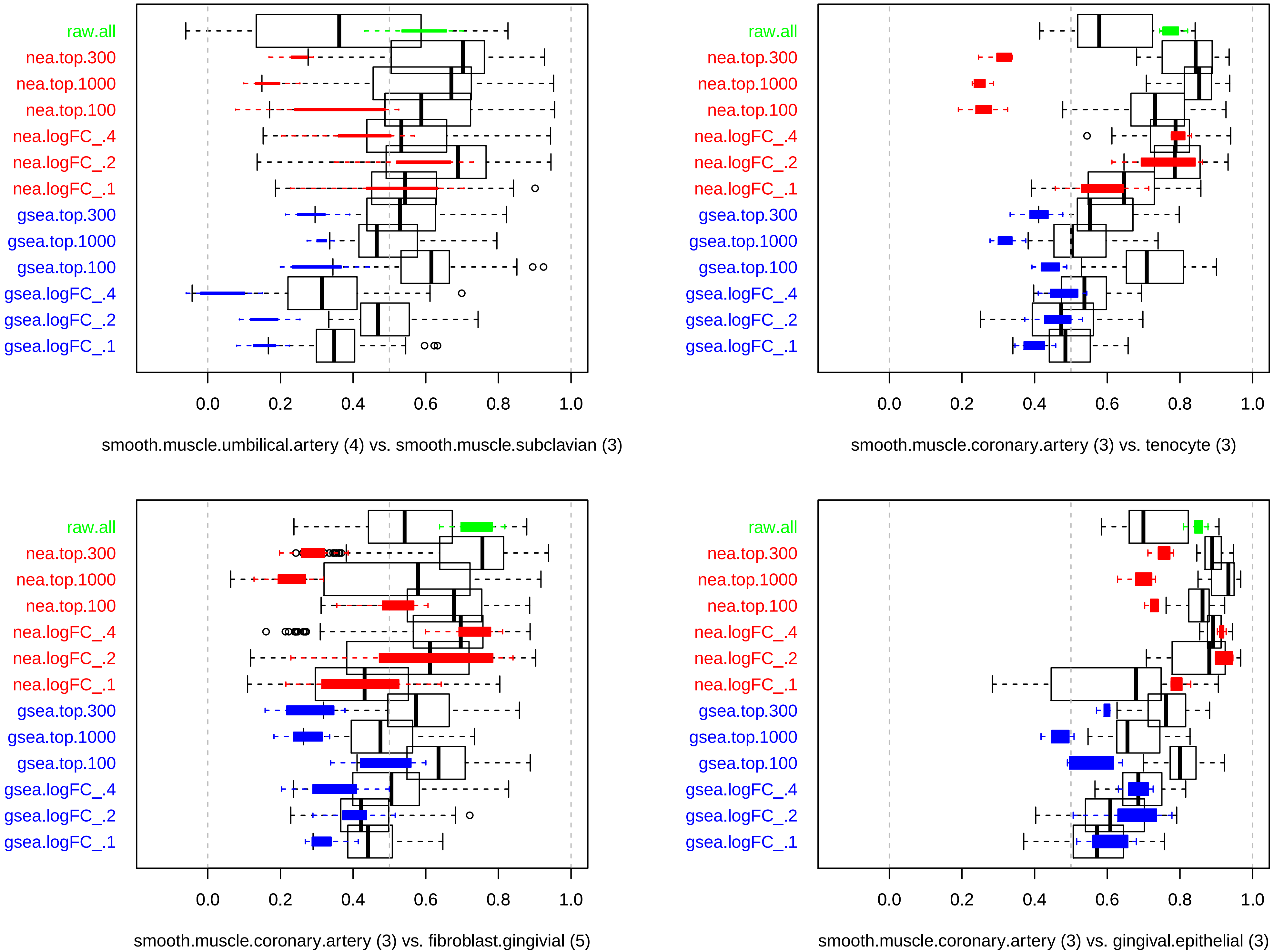
raw.all
nea.top.300
nea.top.1000
nea.top.100
nea.logFC_.4
nea.logFC_.2
nea.logFC_.1
gsea.top.300
gsea.top.1000
gsea.top.100
gsea.logFC_.4
gsea.logFC_.2
gsea.logFC_.1
0.0
0.2
0.4
0.6
0.8
1.0
smooth.muscle.umbilical.artery (4) vs. smooth.muscle.subclavian (3)
smooth.muscle.coronary.artery (3) vs. tenocyte (3)
smooth.muscle.coronary.artery (3) vs. fibroblast.gingivial (5)
smooth.muscle.coronary.artery (3) vs. gingival.epithelial (3)

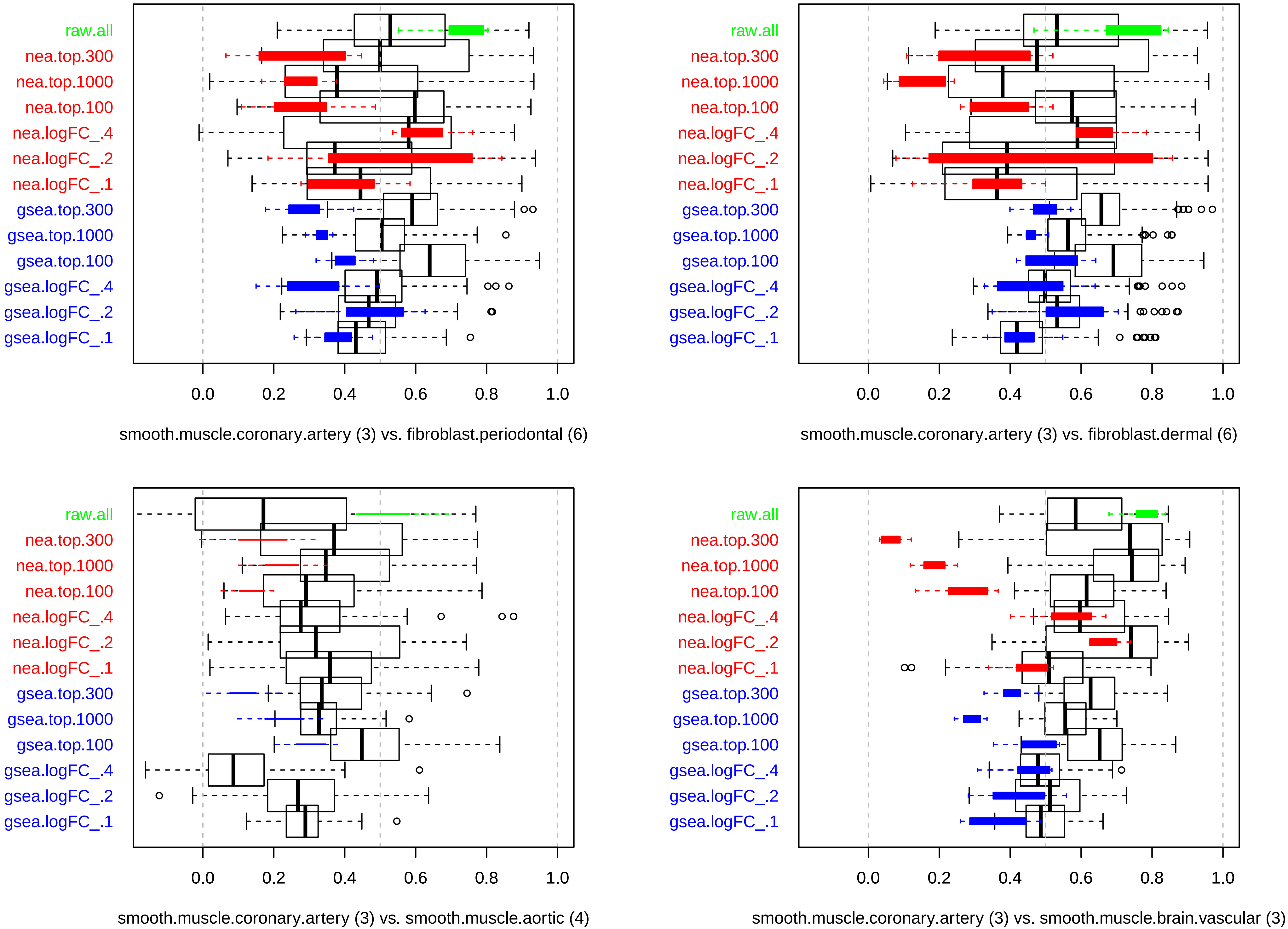

raw.all
nea.top.300
nea.top.1000
nea.top.100
nea.logFC_.4
nea.logFC_.2
nea.logFC_.1
gsea.top.300
gsea.top.1000
gsea.top.100
gsea.logFC_.4
gsea.logFC_.2
gsea.logFC_.1
0.0
0.2
0.4
0.6
0.8
1.0
smooth.muscle.coronary.artery (3) vs. fibroblast.periodontal (6)
smooth.muscle.coronary.artery (3) vs. fibroblast.dermal (6)
smooth.muscle.coronary.artery (3) vs. smooth.muscle.aortic (4)
smooth.muscle.coronary.artery (3) vs. smooth.muscle.brain.vascular (3)

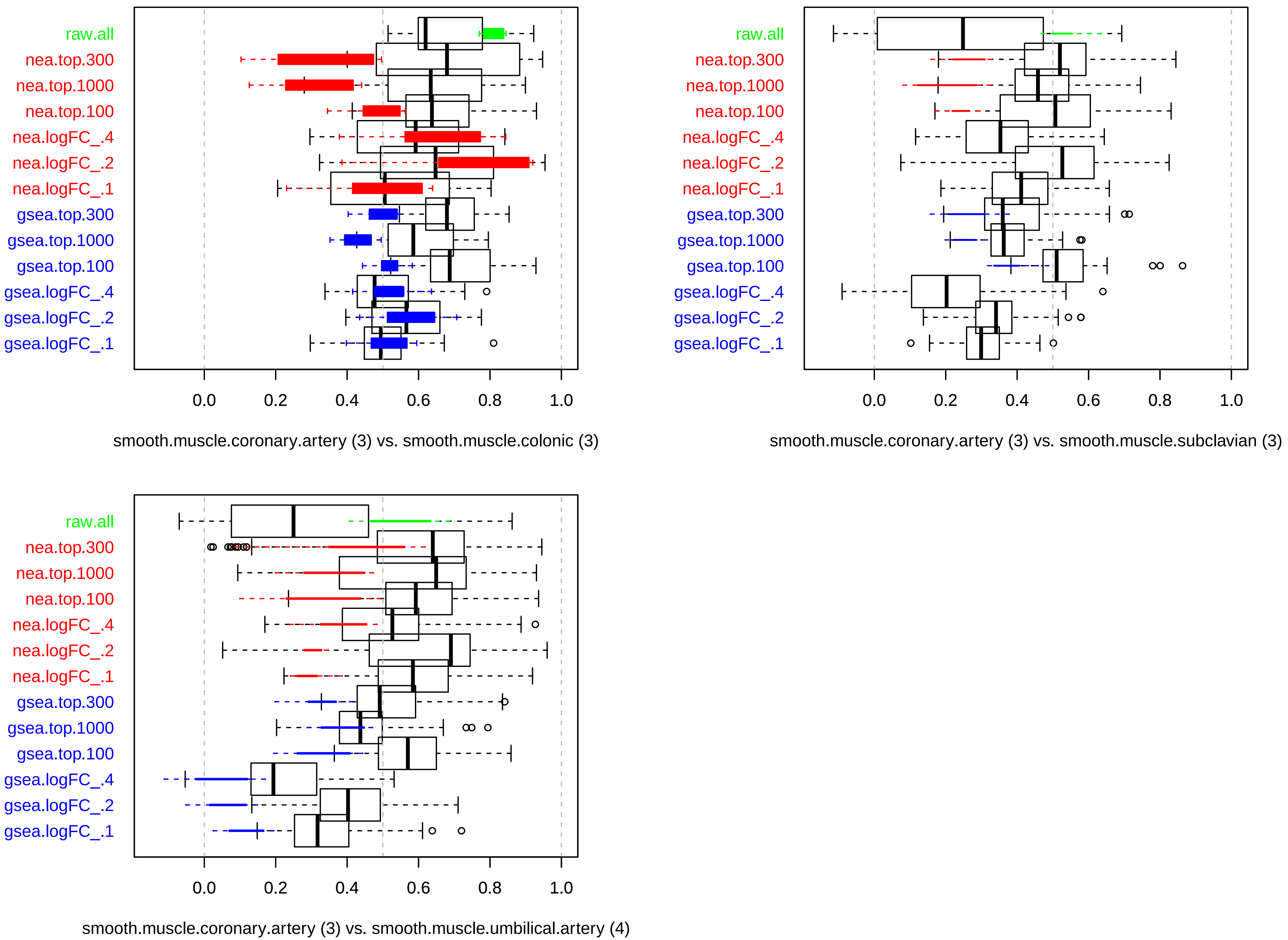
raw.all
nea.top.300
nea.top.1000
nea.top.100
nea.logFC_.4
nea.logFC_.2
nea.logFC_.1
gsea.top.300
gsea.top.1000
gsea.top.100
gsea.logFC_.4
gsea.logFC_.2
gsea.logFC_.1
0.0
0.2
0.4
0.6
0.8
1.0
smooth.muscle.coronary.artery (3) vs. smooth.muscle.colonic (3)
smooth.muscle.coronary.artery (3) vs. smooth.muscle.subclavian (3)
smooth.muscle.coronary.artery (3) vs. smooth.muscle.umbilical.artery (4)
